# Efficacy, User Engagement, and Acceptability of Cognitive Behavioral Therapy–Oriented Psychological Chatbots for Adults With Depressive and/or Anxiety Symptoms: Systematic Review and Meta-Analysis of Randomized Controlled Trials

**DOI:** 10.2196/82677

**Published:** 2026-05-08

**Authors:** Bingyan Gong, Nisha Yao, Hangxin Xie, Chuncheng Huang, Tomoko Kishimoto, Howard Berenbaum, Wenting Mu

**Affiliations:** 1 Department of Psychological and Cognitive Sciences, Tsinghua University, 30 Shuangqing Road, Haidian District, Beijing, 100084, China, 86 15810389613; 2 School of Kinesiology and Health, Capital University of Physical Education and Sports, Beijing, China; 3 Faculty of Psychology, Beijing Normal University, Beijing, China; 4 Department of Psychology, University of Illinois Urbana-Champaign, Champaign, IL, United States

**Keywords:** chatbots, cognitive behavioral therapy, digital mental health, depression, anxiety, systematic review, meta-analysis, engagement, acceptability

## Abstract

**Background:**

Cognitive behavioral therapy (CBT) is the most examined psychotherapy for depression and anxiety, but delivery faces significant barriers such as limited access, cost, and time constraints. CBT-oriented psychological chatbots offer a promising means of addressing these challenges. Yet, their overall efficacy, user engagement, and acceptability have not been systematically synthesized.

**Objective:**

This study aimed to evaluate the efficacy, user engagement, and acceptability of CBT-oriented chatbots for adults with depressive and/or anxiety symptoms.

**Methods:**

A systematic search of 9 databases, including PubMed, Cochrane Central Register of Controlled Trials, Embase, Web of Science, PsycINFO, CINAHL, China National Knowledge Infrastructure, WanFang, and VIP Databases, was conducted from inception to February 2026. Eligibility criteria included randomized controlled trials comparing CBT-oriented chatbots with control groups in adults with depressive and/or anxiety symptoms. Risk of bias (ROB) was assessed using the Cochrane ROB tool. Random-effects meta-analyses (Hartung-Knapp-Sidik-Jonkman adjustment) calculated pooled effect sizes (Hedges *g*), 95% CIs, and 95% prediction intervals (PIs). Heterogeneity was evaluated using the *I*² statistic, and Galbraith plots were used to identify outliers for subsequent sensitivity analyses. Subgroup and meta-regression analyses examined potential moderators. The certainty of evidence was evaluated using the GRADE (Grading of Recommendations Assessment, Development, and Evaluation) approach. Data on user engagement and acceptability were extracted and synthesized using narrative and quantitative methods where available.

**Results:**

Twenty-nine eligible randomized controlled trials were included. CBT-oriented psychological chatbots produced a moderate reduction in depressive symptoms at postintervention (*g*=−0.55, 95% CI −0.70 to −0.40, 95% PI −1.23 to 0.13) and a small reduction in anxiety symptoms (*g*=−0.26, 95% CI −0.37 to −0.14, 95% PI −0.67 to 0.15). At follow-up, effects were small for depression (*g*=−0.32, 95% CI −0.55 to −0.09, 95% PI −0.93 to 0.29) and nonsignificant for anxiety (*g*=−0.19, 95% CI −0.43 to 0.04, 95% PI −0.84 to 0.46). Subgroup and meta-regression analyses revealed that anxiety outcomes were significantly moderated by clinical profiles—showing distinct advantages for comorbid symptoms—and the proportion of female participants. The CBT-oriented chatbots received an adequate level of engagement that complied with digital intervention standards. Although user satisfaction ratings were generally favorable, technical limitations and repetitive interaction patterns remain to be addressed to enhance overall acceptability. Regarding the limitations of evidence, the overall certainty was rated as very low to low, predominantly driven by high ROB and substantial heterogeneity.

**Conclusions:**

This study innovatively isolates CBT-oriented chatbots from broader digital interventions, providing a precise, methodology-driven evaluation of theoretically grounded therapeutics. This review brings critical evidence to the field that these tools yield significant short-term relief, particularly for comorbid anxiety profiles. In the real world, CBT chatbots offer profound potential as scalable, low-barrier first-line tools. To sustain engagement, future developments must evolve from rigid rule-based scripts toward adaptive, large language model–driven architectures while ensuring clinical safety.

## Introduction

Mental disorders have become a growing public health concern worldwide and are among the leading causes of the global health–related burden [1]. The Global Burden of Disease Study 2019 found that depressive disorders accounted for the highest proportion (37.3%) of the global burden of disease attributable to mental disorders, followed by anxiety disorders (22.9%) [2]. In addition to causing considerable suffering and impaired functioning, when severe, these illnesses can be life-threatening [3]. Meanwhile, depressive and anxiety disorders impose substantial economic burden through excessive health care expenditures [4,5]. According to *The Lancet Global Health*, every dollar invested in scaled-up treatment for depression and anxiety yields a 4-fold return in improved health and productivity outcomes [6]. Therefore, there is an urgent imperative to develop effective treatments for depressive and anxiety disorders.

Cognitive behavioral therapy (CBT) is the most examined type of psychotherapy for depression and anxiety [7-9]. However, access to CBT remains limited due to resource constraints, long waiting times, stigma, and a preference for self-help strategies [10-12]. These challenges highlight the need for alternative delivery methods that are more accessible, flexible, and cost-effective. Digital mental health interventions (DMHIs) have emerged as promising, scalable solutions capable of delivering standardized, protocol-adherent CBT. This approach enhances accessibility and cost-effectiveness, thereby addressing the escalating demand for mental health services [13,14]. Among these innovations, chatbots have attracted considerable attention due to their capacity to provide immediate, structured therapeutic support through emulation of human-like dialogue [15].

CBT-oriented psychological chatbots are designed to deliver therapeutic content based on CBT principles, guiding users through processes such as cognitive restructuring, mood monitoring, and behavior activation [16]. Although an increasing number of studies have evaluated CBT-oriented chatbots for depressive and anxiety symptoms, the findings remain inconsistent. Some studies have reported significant reductions in depressive and/or anxiety symptoms [17-28], while others have found no significant improvements compared with control groups [29-42]. Mixed results have also been observed, with some interventions showing effectiveness for depression but not anxiety [43-45]. Given these inconsistencies, a systematic synthesis is needed to clarify the overall efficacy of CBT-oriented chatbots and identify factors that may influence therapeutic outcomes.

To date, no systematic review has specifically focused on the efficacy of CBT-oriented chatbots for addressing depression and/or anxiety. Existing reviews typically encompass a wide spectrum of digital psychological technologies or chatbot interventions in general mental health, rather than isolating the specific efficacy of CBT-based chatbots for depression and/or anxiety. Consequently, their findings reflect the aggregated effects of heterogeneous interventions, making it difficult to draw conclusions about the unique contribution of CBT-oriented chatbots. In addition to this broad scope, existing reviews also suffer from several methodological limitations: (1) lack of quantitative synthesis: 2 reviews have only descriptive summaries without conducting meta-analysis, resulting in a lack of specific numerical support for the results and difficulty in determining the magnitude and statistical significance of the effects [46,47]. (2) Limited statistical power: 3 systematic reviews examine chatbots for mental health improvement, but they included a relatively small number of randomized controlled trials (RCTs) targeting anxiety and/or depression (ranging from 2 to 13), resulting in insufficient statistical power [48-50]. This scarcity is primarily due to the fact that the literature search was conducted before the recent surge in digital health publications. (3) Incomplete outcome assessment: 1 systematic review restricts its quantitative synthesis exclusively to depressive symptom measurements across the 11 eligible trials, while failing to analyze anxiety symptoms [51]. (4) Inclusion of nontarget populations: another review [52] includes individuals who did not exhibit symptoms of anxiety or depression prior to intervention, such as Suharwardy et al [53] and Kleinau et al [54], which could potentially lead to underestimation of the intervention’s true efficacy and even false-negative results. (5) Limited exploration of moderators: only 4 reviews investigate efficacy moderators using subgroup or meta-regression analyses [48,49,51,52]. Consequently, the evidence regarding the efficacy of CBT-oriented chatbots for depressive and/or anxiety symptoms remains fragmented and inadequately synthesized. The underlying mechanisms affecting their efficacy are still unclear.

Beyond therapeutic efficacy, engagement and acceptability are vital in impacting clinical outcomes. Engagement refers to the extent to which a user is exposed to intervention content, which is critical to realizing the promise of digital interventions in the real world [55]. Acceptability refers to the degree to which individuals’ perceived ease of use and perceived usefulness of a technology positively influence their usage intentions, thereby driving its adoption [56]. Nonetheless, prior reviews have not elucidated acceptability and engagement with CBT-oriented chatbots in depressive and anxiety symptoms.

This study is the first to systematically isolate theoretically grounded, CBT-oriented chatbots from the broader landscape of digital conversational agents. The study aimed to (1) evaluate the therapeutic efficacy of CBT-oriented chatbots in adults with depressive and/or anxiety symptoms, (2) systematically explore potential moderators of intervention efficacy, and (3) synthesize evidence on user engagement and acceptability to provide actionable insights for future clinical implementation. Clarifying the therapeutic potential and user experience of CBT-oriented chatbots is critical, given the urgent global demand for accessible digital mental health solutions. These findings establish an evidence base to advance the development and optimization of scalable, evidence-driven chatbots.

## Methods

### Study Design

This systematic review and meta-analysis was conducted in accordance with the PRISMA (Preferred Reporting Items for Systematic Reviews and Meta-Analyses) guidelines [57], and the PRISMA 2020 Expanded Checklist was used. The study protocol was prospectively registered at PROSPERO (CRD42024615506).

### Eligibility Criteria

#### Participants

The included studies involved adults aged 18 years and older with baseline depression and/or anxiety symptom scores meeting or exceeding the defined cutoff for mild severity. Participants were categorized as depression or anxiety-only participants (individuals presenting with symptoms exclusively within the depression and/or anxiety, without documented co-occurring somatic or other psychological symptoms), and participants with comorbid symptoms (individuals with depressive and/or anxiety symptoms accompanied by at least 1 co-occurring somatic symptom or other psychological symptoms).

#### Intervention

Trials were included if they clearly specified the utilization of CBT-oriented psychological chatbots as an intervention modality. CBT-oriented psychological chatbots were defined as systems integrating CBT techniques—such as cognitive restructuring, behavioral activation, exposure, and problem-solving—into chatbot interactions. The chatbots engaged in 2-way communication with users and were delivered via mobile or computer devices. Studies in which chatbots merely served as reminders to encourage user participation, rather than delivering therapeutic interventions, were excluded.

#### Control or Comparison

The studies included at least 1 control condition, active (other active interventions, treatment-as-usual, psychological placebo, and attention control) or passive (waitlist, blank control, or assessment only) control group.

#### Outcomes

The primary outcomes were quantitative measures of depression or anxiety symptom severity. The secondary outcomes included user engagement and acceptability of CBT-oriented psychological chatbots. Engagement in this review was defined as any objective indicator used to quantify the extent of intervention use, including attrition rate, number of logins, module completion, frequency of use, and time spent in the intervention [58]. Acceptability was defined as participants’ psychological and emotional attitudes toward the chatbot interventions, including satisfaction, tolerance, and user experience [56,59].

#### Study Design and Characteristics

The eligible studies were RCTs published in English or Chinese. The review included peer-reviewed papers, dissertations, and conference proceedings, while it excluded preprint papers, conference abstracts, proposals, and editorials. Studies with duplicate published data were excluded.

### Search Strategy

The systematic literature search was designed, conducted, and reported in accordance with the PRISMA-S (Preferred Reporting Items for Systematic Reviews and Meta-Analyses—Search Extension) guideline [60]. The search strategy consisted of 2 steps. First, the following electronic databases were systematically searched from the inception to April 1, 2025, with a final update on February 9, 2026: PubMed (via NCBI), Cochrane Central Register of Controlled Trials (via Wiley), Embase (via Elsevier), Web of Science (via Clarivate), PsycINFO (via APA PsycNet), CINAHL (via EBSCOhost), China National Knowledge Infrastructure, WanFang, and VIP Databases. The search strategy combined MeSH (Medical Subject Headings) and free-text terms related to “depression,” “anxiety,” and “chatbot.” The strategy developed de novo for this review was not adapted from prior searches. No language or date restrictions, nor any predefined search filters, were applied to maximize sensitivity. Second, the reference lists of all eligible studies were manually searched to identify additional relevant studies. No other online resources or study registries were searched. A detailed search strategy is presented as shown in Table S1 in Multimedia Appendix 1.

### Selection Process

EndNote 20 (a reference management software; Clarivate) was used to organize and manage the identified records. After removing duplicates, 2 authors (BG and HX) independently screened all titles or abstracts. Subsequently, the full texts of studies selected in the previous step were independently reviewed by the same 2 authors. The disagreements between the 2 authors were discussed until consensus was reached. When necessary, a third reviewer (WM) was consulted to make a final decision. Interrater reliability, assessed using Cohen κ [61], indicated a very good level of agreement for title or abstract screening (0.86) and full-text review (0.85).

### Data Collection Process

Data extraction was divided into two categories: (1) basic data describing the study characteristics, and (2) core data used for statistical analysis. Two authors (BG and HX) independently coded key study characteristics and extracted relevant outcome data. The corresponding author (WM) or the first author (BG) was contacted via email if information for calculating effect sizes in a study was not reported. If no response was received after 1 month, or if the authors were unable to provide the required data, the study was excluded as unavailable.

### Data Items

Two independent reviewers (BG and HX) extracted the information from eligible studies. The extracted basic information included (1) study characteristics: the name of the first author, the year of publication, country of trial, and study design; (2) participant characteristics: nature of participant, sample size, mean age, and proportion of females; (3) experimental characteristics: intervention group, control group, depression or anxiety measures; and (4) chatbot characteristics: CBT component, intervention duration, number of sessions, duration of each session, follow-up length, chatbot name, platform, chatbot type, dialogue initiative, interaction frequency, input, and output. The core data include (1) primary outcomes: depressive or anxiety symptom severity, and (2) secondary outcomes: engagement and acceptability.

### Risk of Bias Assessment

To assess the risk of bias (ROB) and methodological quality of included studies, 2 rating systems were independently applied by 2 reviewers (BG and HX), with disagreements resolved through discussion and consultation with a third reviewer (WM). The Cochrane Collaboration’s tool for assessing ROB [62] was used to evaluate all studies across 7 domains: “random sequence generation,” “allocation concealment,” “blinding of participants and personnel,” “blinding of outcome assessment,” “incomplete outcome data,” “selective reporting,” and “other bias.” Each domain was rated as having a “low,” “unclear,” or “high risk of bias.”

### Grading the Quality of Evidence

The GRADE (Grading of Recommendations Assessment, Development, and Evaluation) framework was used to evaluate the certainty of evidence provided by this meta-analysis for the different outcomes [63]. The GRADE approach considers 5 domains, including ROB, inconsistency, indirectness, imprecision, and publication bias. Evidence quality was categorized as high, moderate, low, or very low. Two authors (BG and HX) independently evaluated the evidence quality for the following outcomes. Any discrepancies in ratings were resolved through discussion.

### Statistical Analyses

Primary outcome measures were converted into standardized mean differences with 95% CIs and 95% prediction intervals (PIs). When SDs were not available, they were estimated through mathematical transformation [64]. If the SD_change_ or the correlation between pre- and postintervention scores was not reported and could not be calculated, we estimated the SD_change_ using imputed correlation coefficients (*r*) derived from studies that reported complete data. Considering potential variations in temporal stability and clinical constructs, *r* values were calculated separately for depressive symptoms (Post: *r*=0.85; Follow-up: *r*=0.73) and anxiety symptoms (Post: *r*=0.67; Follow-up: *r*=0.67). If both intention-to-treat and completer analyses were presented, data from the former were extracted and analyzed. For studies with multiarm designs that included multiple experimental or control groups, we extracted the data from the group most relevant to our research questions. If multiple groups met the inclusion criteria, we combined the means and SDs from these arms, as suggested by the Cochrane guidelines for integrating multiple groups from a single study (Table S2 in Multimedia Appendix 1 [20,23,25,27,35]). When a study had multiple follow-up data, we extracted the longest follow-up for analysis [64]. Given potential baseline differences, the change from baseline scores was computed for each group to represent efficacy. The effect sizes for depressive and anxiety symptoms were calculated separately, using Hedges *g* to adjust for small sample bias, with 95% CI and *z* statistics to determine significance (*P*<.05). Following Cohen’s [65] (1988) conventions, effect sizes (Hedges’ *g*) of 0.2, 0.5, and 0.8 were interpreted as small, medium, and large, respectively. All meta-analyses were performed using an inverse variance random-effects model with Hartung-Knapp-Sidik-Jonkman adjustment [66].

Heterogeneity was assessed using Cochrane Q test (*P*<.10 for significance) and the *I*^2^ statistics, with interpretation thresholds following the Cochrane Handbook [64]. Specifically, an *I*^2^ of 0%-40% was considered to represent unimportant heterogeneity, 30%-60% moderate heterogeneity, 50%-90% substantial heterogeneity, and 75%-100% considerable heterogeneity [64]. To visually explore potential sources of heterogeneity and identify specific outlier studies, a Galbraith plot was generated. Studies positioned outside the 95% confidence bounds of the regression line were classified as outliers. Subsequently, a sensitivity analysis excluding these identified outliers was conducted to evaluate the robustness of the pooled effect sizes and to maintain overall homogeneity [64]. To explore potential moderating factors and identify sources of heterogeneity, subgroup analyses and meta-regression for the primary outcomes (depressive and anxiety symptoms) were conducted. The investigated covariates included participants’ symptom profiles, control conditions, intervention duration, delivery platform, chatbot type, country, and ROB. Spearman correlation analyses showed no multicollinearity among moderators (|r| <0.7; Table S3 in Multimedia Appendix 1). To examine the relative importance of different moderator domains, we adopted a multimodel inference approach using information criteria. We compared the 3 prespecified models (model 1: sample characteristics, model 2: technical features, and model 3: study design) using the Akaike information criterion (AIC) [67] and the Bayesian information criterion (BIC) [68]. Lower AIC and BIC values indicate a more parsimonious model that achieves a better trade-off between goodness of fit and complexity [67,68]. Small-study effects were assessed through visual inspection of funnel plot asymmetry and the Egger test [69], which was applied when 10 or more trials were available [70]. For all statistical analyses, a significance level of α<.05 was considered. All analyses were conducted using Stata SE (version 17.0; StataCorp LLC).

Significant heterogeneity was observed across studies in the reporting of chatbot engagement and acceptability. The variability manifested in several key dimensions: (1) diverse operational definitions of engagement (eg, adherence rate, session frequency, and number of messages exchanged) and acceptability (eg, satisfaction and subjective user experience), (2) heterogeneous measurement instruments (eg, standardized questionnaires, study-designed items, and system usage logs), and (3) inconsistent reporting of outcome metrics (eg, means, SDs; medians; proportions; and only descriptive summaries). This variability in measurement and reporting precluded the calculation of comparable effect sizes across studies, making a meta-analysis unfeasible. Therefore, findings related to engagement and acceptability were qualitatively summarized for each included trial.

## Results

### Study Selection

**Figure 1.**
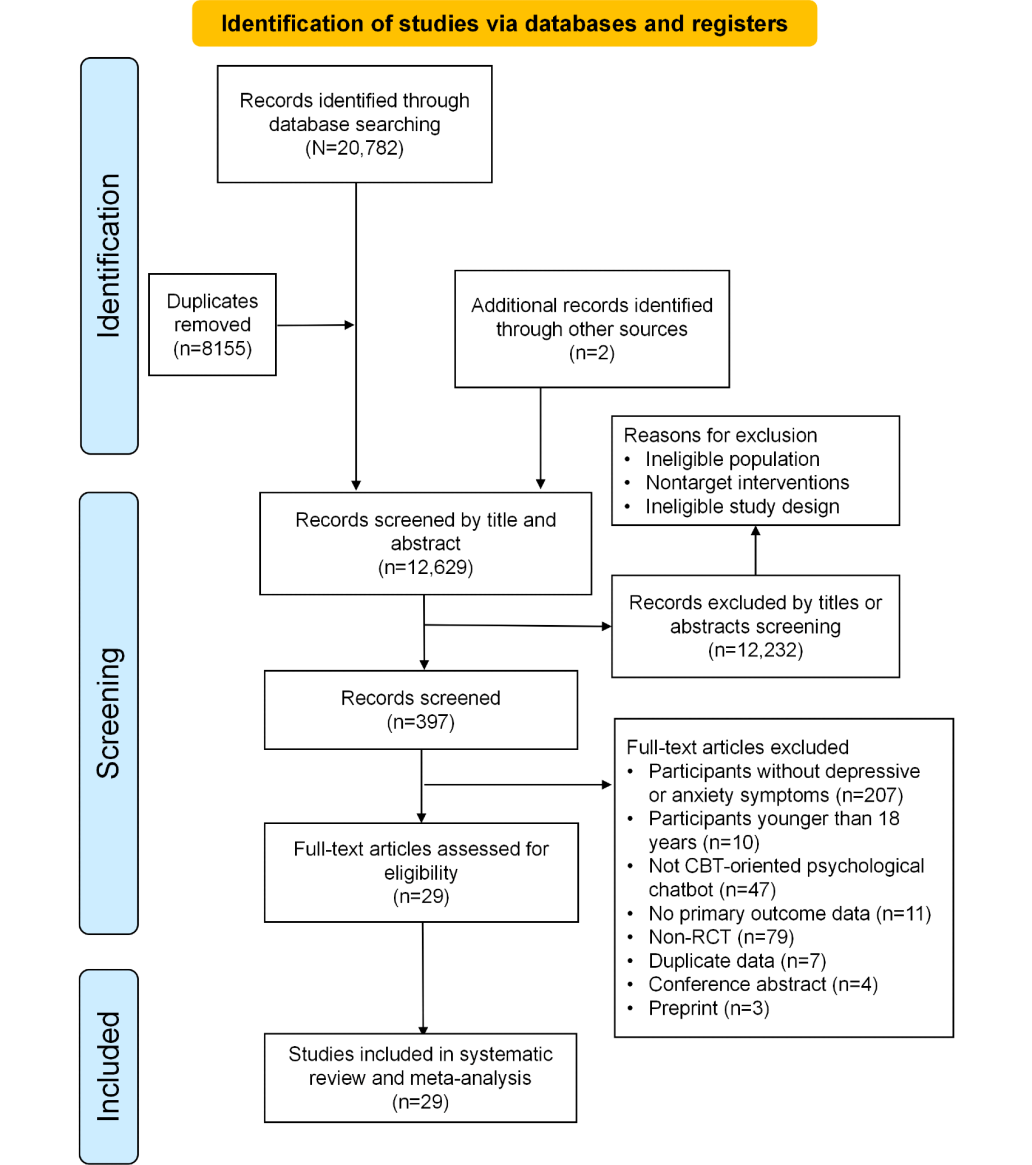
PRISMA (Preferred Reporting Items for Systematic Reviews and Meta-Analyses) flow diagram. Search and study selection process. CBT: cognitive behavioral therapy; RCT: randomized controlled trial.

The database search yielded 20,782 records and a further 2 records were identified after searching the reference lists of relevant systematic reviews. After removing 8155 duplicates, 12,629 titles or abstracts were assessed for eligibility and 12,232 records were excluded. Following full-text screening, 397 papers were further screened and 368 were excluded. Finally, 29 studies were included in the systematic review and meta-analysis (Figure 1).

### Study Characteristics

The key characteristics of studies and participants are summarized in Table S4 in Multimedia Appendix 1 [17-45]. The studies were published between 2016 and 2026. Nine trials were from the United States [24,27,29,36,38,40,41,44,45], 7 from China [19,20,23,25,26,30,31], 2 from Canada [21,33], 2 from South Korea [37,39], 2 from Jordan [17,32], 1 from the United Kingdom [18], 1 from Switzerland [43], 1 from Poland [34], 1 from Italy [35], 1 from Argentina [42], 1 from Switzerland, Germany, and Austria [22], and 1 from Romania, Spain, and Scotland [40]. Among these trials, 24 trials were 2-arm RCTs [17-19,21,22,24,26,28-34,36-45], 3 trials were 3-arm RCTs [23,25,27], 1 trial was a 4-arm RCT [35], and 1 trial was a 5-arm RCT [20]. Sixteen studies used active controls [17,18,20,25-29,31,32,34,37,39,40,42,45], and 13 studies used passive controls [19,21-24,30,33,35,36,38,41,43,44]. All studies were published in English.

Half of the studies (15/29, 52%) were conducted in only depression or anxiety participants [18,19,21,23-30,34,40,42,45], while the remainder (14/29, 48%) involved participants with comorbid symptoms (eg, arthritis or diabetes, frequent headaches, irritable bowel syndrome, stress symptoms, and attention deficit) [17,20,22,31-33,35-39,41,43,44]. The included studies reported data from a total of 5686 participants. Study sample sizes ranged from 18 to 1489. The average age ranged from 18.9 to 56.5 years, and the proportion of female participants ranged from 36.7% to 100%. The most commonly used measure of depression severity among the included studies was the Patient Health Questionnaire-9 (17/29) [18,20-23,25-27,29-31,33,34,36,42,43,45]. For anxiety severity, the most commonly used measure was the Generalized Anxiety Disorder-7 (17/29) [18-23,26,27,29,30,33,35,38,41-43,45]. Regarding the statistical approach for handling missing data, 13 of the 27 included RCTs used an intention-to-treat analysis [17-20,23,25,26,28-30,35,37,39], while the remaining 16 trials reported results based on completer analysis [21,22,24,27,31-34,36,38-43,45].

Intervention and chatbot characteristics are shown in Table S5 in Multimedia Appendix 1 [17-45]. In the included studies, multiple CBT intervention components for psychological improvement were involved. Psychoeducation provides knowledge about psychological processes and therapeutic principles. Cognitive strategies identify and challenge biases or distortions, often using methods such as the ABC model and cognitive restructuring to modify maladaptive thoughts. Behavioral techniques, including behavioral activation, exposure, and behavioral experiments, were used to adjust maladaptive behavior patterns, increase engagement in valued activities, and reduce avoidance. Emotion-focused approaches targeted the identification, understanding, and regulation of emotions. Problem-solving therapy helps participants address life challenges by enhancing their problem-solving skills. Other components involve sleep interventions, relaxation, mindfulness, gratitude practices, and self-monitoring.

Five studies recommended that participants interact freely with the chatbot [21,22,26,34,39], while 13 studies required daily access [19,20,23,25,27,28,30,31,36-38,44,45]. The duration of chatbot interventions varied considerably, ranging from 1 week to 16 weeks. Eleven studies reported follow-up outcomes: 5 provided follow-up assessments at 1 month [20,23,25,30,34], 2 at 8 weeks [18,44], 3 at 3 months [17,35,36], and 1 at both 3 and 6 months [41]. More than half of the studies (n=16) implemented chatbots as independent, stand-alone systems (eg, MISHA, Wysa, Minder, and TEO) [18-22,28,29,33,35-40,43,45], while 13 studies deployed chatbots within multimodel platform (eg, Website, Facebook message, WeChat, and Amazon’s Alexa) [17,23-27,30-32,34,41,42,44]. Thirteen studies used artificial intelligence (AI)–based chatbots that use machine learning, natural language processing, or other AI technologies to interpret users’ input and generate responses [19,24-29,33-35,38,42,45]. In contrast, 16 studies implemented rule-based chatbots, where responses were determined by predefined rules or decision trees [17,18,20-23,30-32,36,37,39-41,43,44]. Chatbots led and controlled the conversation in 58.6% (17/29) of the studies [17,20,22-25,27,28,32,33,36,38,41-45]. The input modalities for participants included written language and/or emoji (26/29, 90%) [17,19-23,25,27-45], spoken language only (1/23, 4%) [24], or a combination of written and spoken languages (2/29, 7%) [18,26]. The chatbot output mainly consisted of text (7/29, 24%) [18,23,26,30,33,35,36], voice and text (1/29, 3%) [24], text and emoji (3/29, 10%) [27,34,42], text and/or voice combined with image (2/29, 7%) [25,29], and text combined with other modalities such as audio, video, emojis, images, and infographics (16/29, 55%) [17,19-22,28,31,32,37-41,43-45].

### Risk of Bias

**Figure 2.**
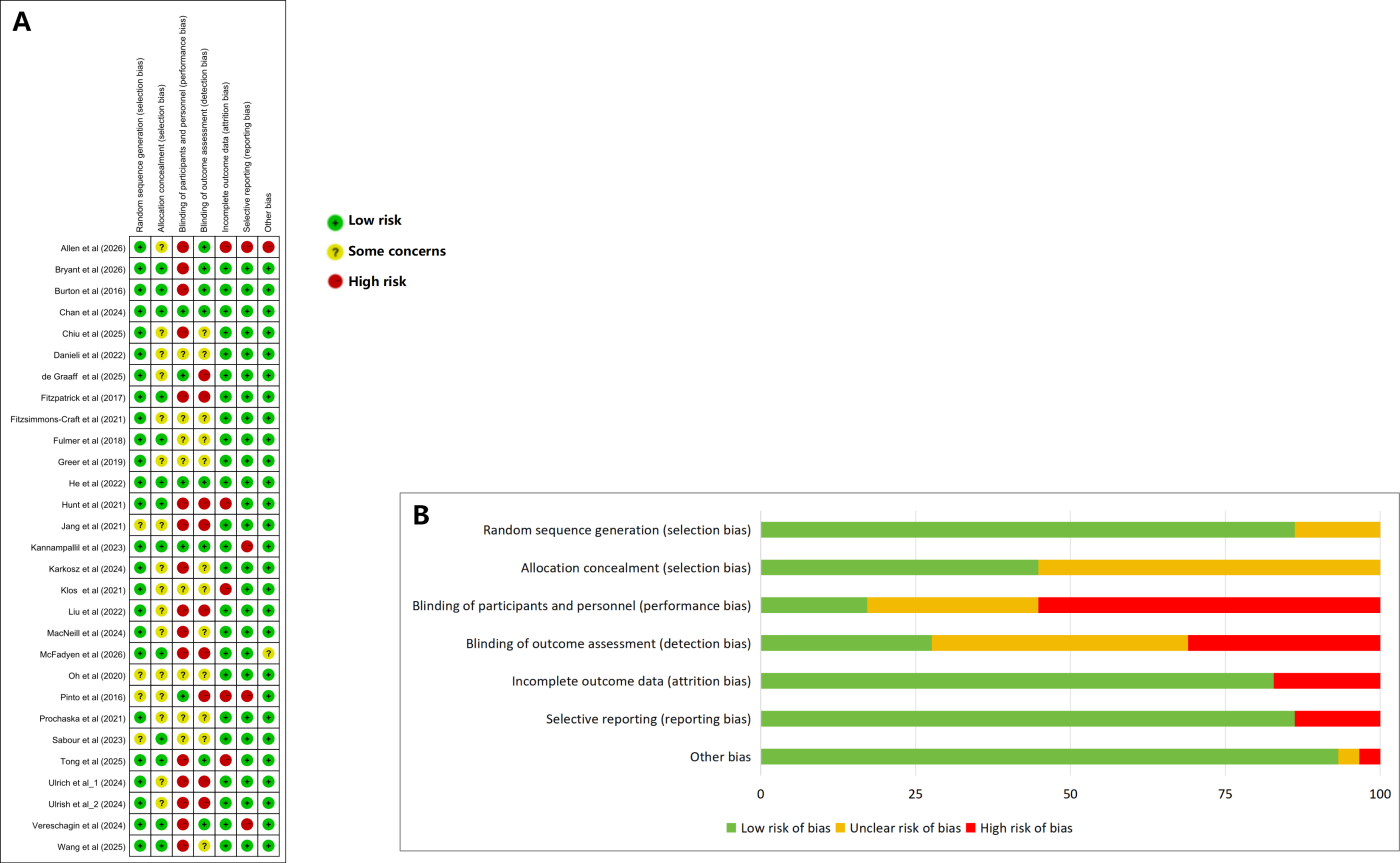
The results of the risk of bias assessment of included studies [17-45]. (**A**) Risk of bias summary and (**B**) risk of bias graph.

Twenty-five studies [17-22,24-36,38,41-45] described adequate random sequence generation, indicating low risk. Allocation concealment was unclear in 16 studies [18,22,26,31-35,37-44]. Five studies [18,30,36,40,42] had an ROB arising from incomplete outcome data, and 4 studies [18,21,24,40] exhibited risk concerning selective reporting. The most prevalent issue involved blinding implementation. Sixteen studies [17-19,21,22,26,28-31,33,34,36,37,43,45] exhibited high ROB in the blinding of participants and personnel, while 9 studies [22,26,29,32,36,37,40,43,45] showed high risk in blinding of outcome assessment. These findings clearly indicate that achieving adequate blinding of participants and personnel poses a particular challenge in psychotherapy research. The ROB graph and ROB summary are shown in Figures 2A and 2B [17-45].

### Quality of Evidence

According to the GRADE summary of findings, the main limitations included the high RoB, the substantial heterogeneity, and the imprecision among the RCTs. As a result, the certainty of evidence was rated as low for postintervention depressive and anxiety symptoms, as well as for long-term depressive symptoms, and very low for long-term anxiety symptoms (Table S6 in Multimedia Appendix 1).

### Efficacy

#### Depressive Symptoms

**Figure 3.**
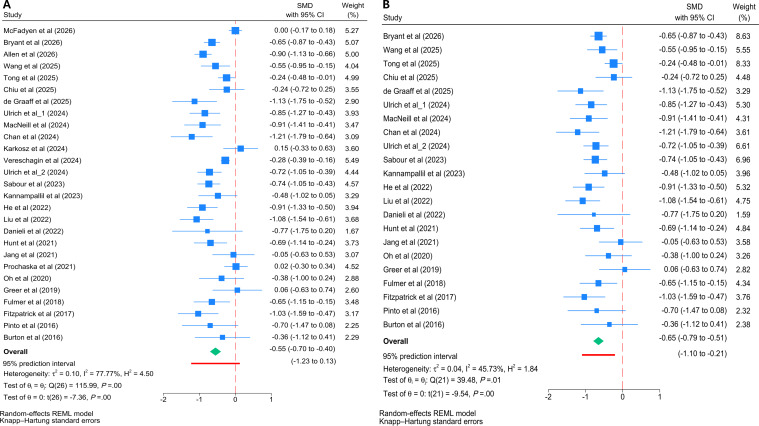
Forest plots of the efficacy of cognitive behavioral therapy–oriented chatbots on depressive symptoms at postintervention. (**A**) Primary meta-analysis of all included trials and (**B**) sensitivity analysis excluding 5 outlier studies. The green diamonds represent the pooled overall effect size and its 95% CI. The thick red horizontal lines placed directly underneath the summary diamonds represent the 95% prediction interval. REML; SMD: standardized mean difference [17-40,43-45].

Meta-analyses of 27 trials (4247 participants) [17-40,43-45] demonstrated significant effects of chatbot-based interventions decreasing postintervention depressive symptom scores (*I*^2^=77.8%; *P*<.001), with a medium effect size (*g*=−0.55, 95% CI −0.70 to −0.40, 95% PI −1.23 to 0.13; Figure 3A). No evidence of significant small-study effects was found by the Egger test (*z*=−0.96, *P*=.335), and visual inspection of the funnel plot confirmed symmetry (Figure S1 in Multimedia Appendix 2). To explore potential sources of heterogeneity, a Galbraith plot was generated, identifying 5 outlier studies [18,21,29,34,38] (Figure S2 in Multimedia Appendix 2). A sensitivity analysis excluding the 5 studies revealed a substantial reduction in interstudy heterogeneity, with *I*² dropping from 77.8% to 45.7%. The repooled analysis demonstrated a slightly stronger and highly consistent medium effect size (*g*=−0.65, 95% CI −0.79 to −0.51). Notably, the 95% PI (−1.10 to −0.21) no longer crossed zero after the exclusion (Figure 3B). These findings not only verify the strong robustness of the primary results but also suggest a more consistent expected efficacy of chatbot-based interventions across future settings.

**Figure 4.**
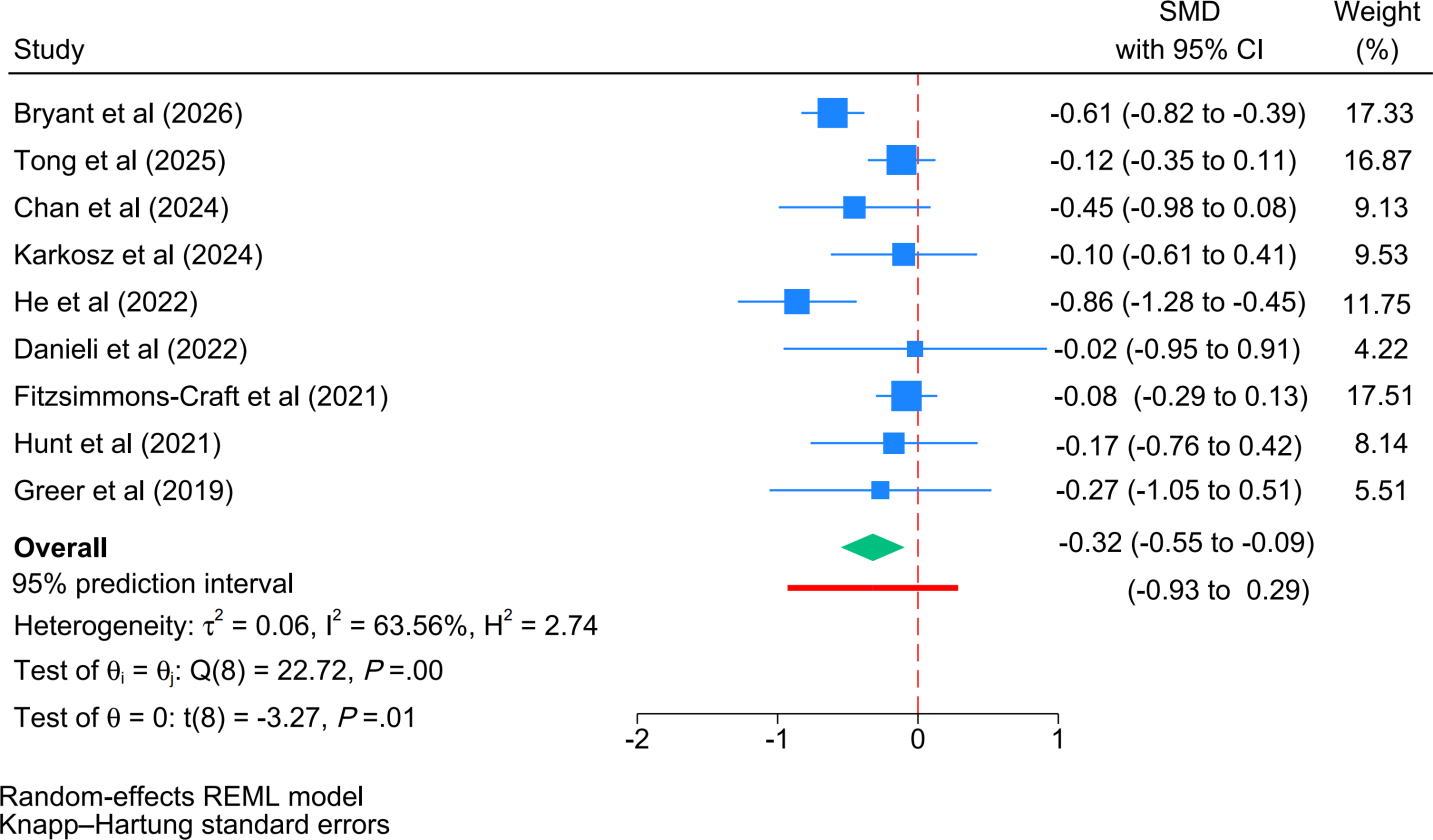
Forest plot of the long-term efficacy (follow-up) of cognitive behavioral therapy–oriented chatbots on depressive symptoms. The green diamond represents the pooled overall effect size and its 95% CI. The thick red horizontal line placed directly underneath the summary diamond represents the 95% prediction interval. REML: restricted maximum likelihood; SMD: standardized mean difference [17,20,25,30,34-36,41,44].

A total of 9 trials (1283 participants) [17,20,25,30,34-36,41,44] were included in the meta-analysis of long-term efficacy on depressive symptoms. The pooled effect size demonstrated a significant improvement favoring chatbot interventions over control conditions on depressive symptoms (*I*^2^=63.6%, *P*=.004), representing a small effect size (*g*=−0.32, 95% CI −0.55 to −0.09, 95% PI −0.93 to 0.29; Figure 4). However, these results should be interpreted with caution, as the small number of trials may limit the statistical power of this follow-up analysis.

#### Anxiety Symptoms

**Figure 5.**
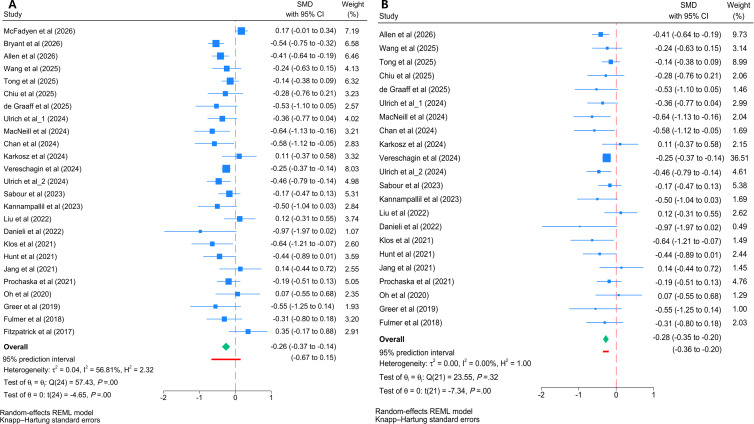
Forest plots of the efficacy of cognitive behavioral therapy–oriented chatbots on anxiety symptoms at postintervention. (**A**) Primary meta-analysis of all included trials and (**B**) sensitivity analysis excluding 3 outlier studies. The green diamonds represent the pooled overall effect size and its 95% CI. The thick red horizontal lines placed directly underneath the summary diamonds represent the 95% prediction interval. REML: restricted maximum likelihood; SMD: standardized mean difference [17-24,26,27,29-39,42-45].

Meta-analyses of 25 trials (4158 participants) [17-24,26,27,29-39,42-45] demonstrated significant effects of chatbot-based interventions in decreasing postintervention anxiety symptom scores (*I*^2^=56.8%, *P*<.001), with a small effect size (*g*=−0.26, 95% CI −0.37 to −0.14, 95% PI −0.67 to 0.15; Figure 5 [17-24,26,27,29-39,42-45]). No significant small-study effects were indicated by the Egger test (*z*=−0.92, *P*=.356), and visual inspection of the funnel plot confirmed symmetry (Figure S1 in Multimedia Appendix 2). To explore potential sources of heterogeneity, a Galbraith plot was generated, identifying 3 outlier studies [17,29,45] (Figure S2 in Multimedia Appendix 2). A sensitivity analysis excluding the 3 studies revealed a substantial reduction in interstudy heterogeneity, with *I*² dropping from 56.8% to 0.0%. The repooled analysis demonstrated a slightly stronger and highly consistent small effect size (*g*=−0.28, 95% CI −0.35 to −0.20). Notably, the 95% PI (−0.36 to −0.20) no longer crossed zero after the exclusion (Figure 5). These findings not only verify the strong robustness of the primary results but also suggest a more consistent expected efficacy of chatbot-based interventions across future settings.

**Figure 6.**
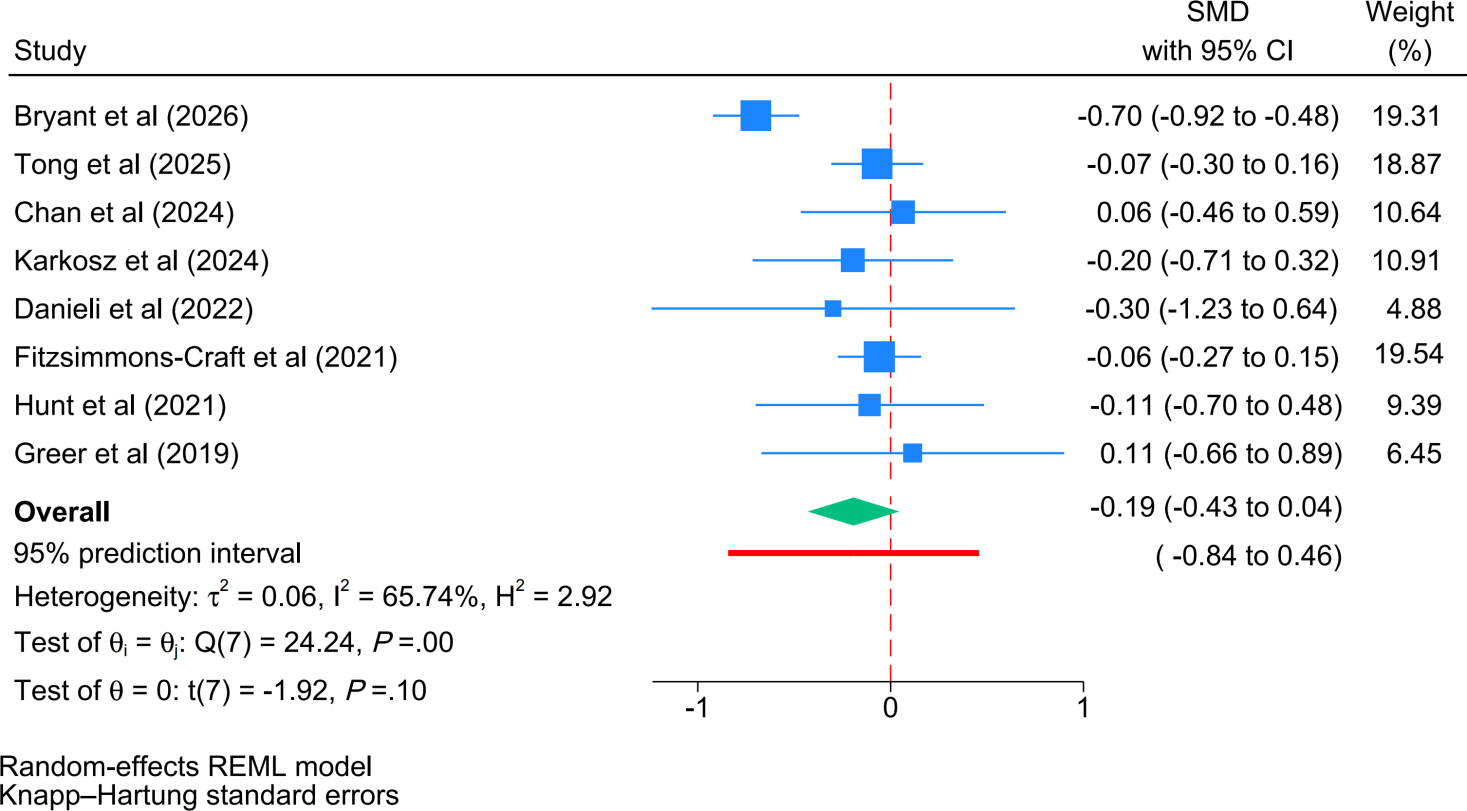
Forest plot of the long-term efficacy (follow-up) of cognitive behavioral therapy–oriented chatbots on anxiety symptoms. The green diamond represents the pooled overall effect size and its 95% CI. The thick red horizontal line placed directly underneath the summary diamond represents the 95% prediction interval. REML: restricted maximum likelihood; SMD: standardized mean difference [17,20,30,34-36,41,44].

A total of 8 trials (1185 participants) [17,20,30,34-36,41,44] were included in the meta-analysis of long-term efficacy on anxiety symptoms. The pooled effect size demonstrated no significant improvement of chatbot interventions over control conditions on follow-up anxiety symptoms (*I*^2^=65.74%, *P*=.001), representing a nonsignificant effect (*g*=−0.19, 95% CI −0.43 to 0.04, 95% PI −0.84 to 0.46; Figure 6 [17,20,30,34-36,41,44]). Given the limited number of studies, these findings should be viewed with caution due to potential constraints on statistical power.

### Moderators of Efficacy

#### Overview

Subgroup analyses were conducted to explore between-study variance and provide a more nuanced understanding of the efficacy of chatbot interventions. The pooled standardized mean difference for each subgroup can be found in Table 1. Meta-regressions were conducted to assess whether age and proportion of females influenced the short-term effect size of CBT-oriented chatbots on depressive and anxiety symptoms. Additionally, to assess the relative importance of these moderators, AIC- or BIC-based multimodel inference was conducted across 3 predefined models (Table S7 in Multimedia Appendix 1 ).

**Table 1.** Subgroup analyses for depressive and anxiety symptoms.

	Number of studies	Heterogeneity		Meta-analysis		
		*I*^2^ (%)	*P* value	*g*	95% CI	*P* value
Total depressive symptoms: EOT^a^						
Participants symptom profiles						.80
Depression or anxiety-only	14	82.99	<.001	−0.54	−0.76 to −0.31	
Comorbid symptoms	13	67.65	<.001	−0.57	−0.82 to −0.32	
Control condition						.46
Active control	15	79.13	<.001	−0.60	−0.85 to −0.35	
Passive control	12	70.05	<.001	−0.49	−0.69 to −0.29	
Intervention duration						.40
1-4 weeks	15	74.03	<.001	−0.49	−0.69 to −0.29	
5-8 weeks	9	84.17	<.001	−0.61	−0.95 to −0.28	
9-16 weeks	3	34.43	.249	−0.78	−1.59 to 0.03	
Platform						.94
Multimodel	11	73.50	<.001	−0.55	−0.82 to −0.27	
Stand-alone	16	80.00	<.001	−0.56	−0.76 to −0.35	
Chatbot type						.64
Rule-based	13	77.37	<.001	−0.59	−0.81 to −0.37	
AI^b^-based	14	72.80	<.001	−0.52	−0.76 to −0.27	
Country						.32
Western	16	80.33	<.001	−0.49	−0.70 to −0.28	
Eastern	11	68.05	.001	−0.64	−0.89 to −0.39	
ROB^c^						.11
Low risk	4	33.34	.178	−0.78	−1.23 to −0.32	
Unclear	10	61.72	.004	−0.39	−0.66 to −0.12	
High risk	13	83.81	<.001	−0.59	−0.83 to −0.36	
Total depressive symptoms: FU^d^						
Follow-up duration						.75
<3 months	5	59.56	.036	−0.36	−0.77 to 0.06	
≥3 months	4	70.57	.006	−0.28	−0.74 to 0.17	
Total anxiety symptoms: EOT						
Participants symptom profiles						*.008^e^*
Depression or anxiety-only	12	64.70	.001	−0.15	−0.32 to 0.02	
Comorbid symptoms	13	5.88	.387	−0.41	−0.54 to −0.28	
Control condition						.37
Active control	13	68.86	<.001	−0.18	−0.39 to 0.02	
Passive control	12	0.00	.535	−0.28	−0.37 to −0.19	
Intervention duration						.40
1-4 weeks	13	5.16	.162	−0.23	−0.34 to −0.12	
5-8 weeks	10	66.81	<.001	−0.38	−0.59 to −0.17	
9-16 weeks	2	69.13	.072	−0.17	−4.13 to 3.79	
Platform						.71
Multimodel	11	41.17	.074	−0.29	−0.46 to −0.11	
Stand-alone	14	66.76	<.001	−0.24	−0.42 to −0.07	
Chatbot type						.13
Rule-based	13	26.30	.257	−0.33	−0.44 to −0.23	
AI-based	12	59.68	.003	−0.16	−0.39 to 0.07	
Country						.67
Western	15	66.34	<.001	−0.29	−0.46 to −0.12	
Eastern	10	42.98	.063	−0.24	−0.42 to −0.06	
ROB						*.02*
Low risk	2	0.00	.882	−0.55	−0.74 to −0.35	
Unclear	10	0.00	.445	−0.24	−0.40 to −0.07	
High risk	13	69.43	<.001	−0.22	−0.40 to −0.04	
Total anxiety symptoms: FU						
Follow-up duration						.24
<3 months	4	0.00	.876	−0.06	−0.21 to 0.09	
≥3 months	4	77.40	<.001	−0.32	−0.84 to 0.21	

a EOT: end of treatment.

b AI: artificial intelligence.

c ROB: risk of bias.

d FU: follow up.

e Italicized values are significant at *P*<.05.

#### Depressive Symptoms

No significant between-subgroup differences on depressive symptoms were observed across all subgroup factors (all *P*>.05). For participants’ symptom profiles, both adults with depression or anxiety only and those with comorbid symptoms showed significant improvements in depression, with moderate effects (*g*=−0.54 and −0.57, respectively). Under control conditions, interventions compared with an active control group yielded a larger effect (*g*=−0.60) than those compared with a passive control (*g*=−0.49). Regarding intervention duration, effects were strongest for interventions lasting 9‐16 weeks (*g*=−0.78), followed by 5‐8 weeks (*g*=−0.61), and 1‐4 weeks (*g*=−0.49). In terms of platform types, both multimodel and stand-alone platform groups significantly affected depression, with effect sizes of −0.55 and −0.56, respectively. For chatbot types, rule-based and AI-based groups showed similar effect sizes (*g*=−0.59 and −0.52, respectively). Regarding the study location, interventions conducted in Eastern countries showed a larger effect size (*g*=−0.64) than those conducted in Western countries (*g*=−0.49). In terms of ROB, studies with a low risk demonstrated the largest effect (*g*=−0.78), followed by those with a high risk (*g*=−0.59) and an unclear risk (*g*=−0.39). Meta-regression analyses suggested that neither the mean sample age (*β*=.003, *P*=.715) nor the proportion of females (*β*=.002, *P*=.804) had a significant impact on depressive symptoms (Figure S3 in Multimedia Appendix 2).

For studies with a follow-up period of less than 3 months, a small but nonsignificant effect was observed (*g*=−0.36, 95% CI −0.77 to 0.06). Similarly, for studies with a follow-up period of 3 months or longer, the effect size was also not statistically significant (*g*=−0.28, 95% CI −0.74 to 0.17). Furthermore, no significant difference was observed between these 2 follow-up durations (*P*=.746). This suggests that intervention effects were not statistically significant at follow-up, regardless of whether the duration was shorter or longer than 3 months.

#### Anxiety Symptoms

Significant between-subgroup differences on anxiety symptoms were observed for participants’ symptom profiles (*P*=.008) and ROB (*P*=.022), while no significant differences were found across the other subgroup factors (all *P*>.05). As for participants’ characteristics, a larger effect size was observed in adults with comorbid symptoms (*g*=−0.41) than in adults with depression or anxiety-only symptoms (*g*=−0.15). Under control conditions, the therapeutic effect of chatbots was more pronounced for passive control groups (*g*=−0.28) in contrast to active control groups (*g*=−0.18). Regarding intervention characteristics, the effect was stronger for interventions lasting 5‐8 weeks (*g*=−0.38) than for interventions lasting 1‐4 weeks (*g*=−0.23) or 9‐16 weeks (*g*=−0.17). Additionally, interventions delivered through multimodal platforms yielded a slightly larger effect (*g*=−0.29) than those using stand-alone platforms (*g*=−0.24). Similarly, the rule-based group showed a marginally larger effect size (*g*=−0.33) compared with the AI-based group (*g*=−0.16). Regarding the study location, interventions conducted in Western countries showed a slightly larger effect (*g*=−0.29) than those in Eastern countries (*g*=−0.24). For ROB, studies with a low risk demonstrated the largest effect (*g*=−0.55), followed by those with an unclear risk (*g*=−0.24) and a high risk (*g*=−0.22). Meta-regression analyses suggested that mean sample age (*β*=−.006, *P*=.394) had no impact on anxiety symptoms. However, a negative association was identified between the proportion of female participants and the pooled effect size (*β*=−.014, *P*=.022), with a higher proportion of females associated with a larger absolute effect size (Figure S3 in Multimedia Appendix 2). Subgroup analysis by follow-up length showed that chatbot interventions had no significant long-term effect on anxiety symptoms, whether the follow-up period was less than 3 months (*g*=−0.06, 95% CI −0.21 to 0.09) or 3 months and longer (*g*=−0.32, 95% CI −0.84 to 0.21). Additionally, there was no significant difference observed between these 2 follow-up durations (*P*=.237).

To explore the importance of moderators, we conducted a multimodal inference analysis using information criteria. For depressive symptoms, the model including study design characteristics (model 3: control type, country, and ROB) emerged as the best-fitting model. It explained the highest explained variance (*R*^2^=16.96%) and yielded the lowest AIC (33.30) and residual heterogeneity (τ²=0.08, *I*²=70.19%). For anxiety symptoms, model 3 (study design) again demonstrated the best fit, with the highest *R*^2^ (63.53%), lowest AIC (8.16), and lowest residual heterogeneity (τ²=0.01, *I*²=28.32%). Notably, all 3 models explained substantially more variance in anxiety symptoms (*R*^2^ range: 39.52%-63.53%) than in depressive symptoms (*R*^2^ range: 4.26%-16.96%).

### Engagement

**Figure 7.**
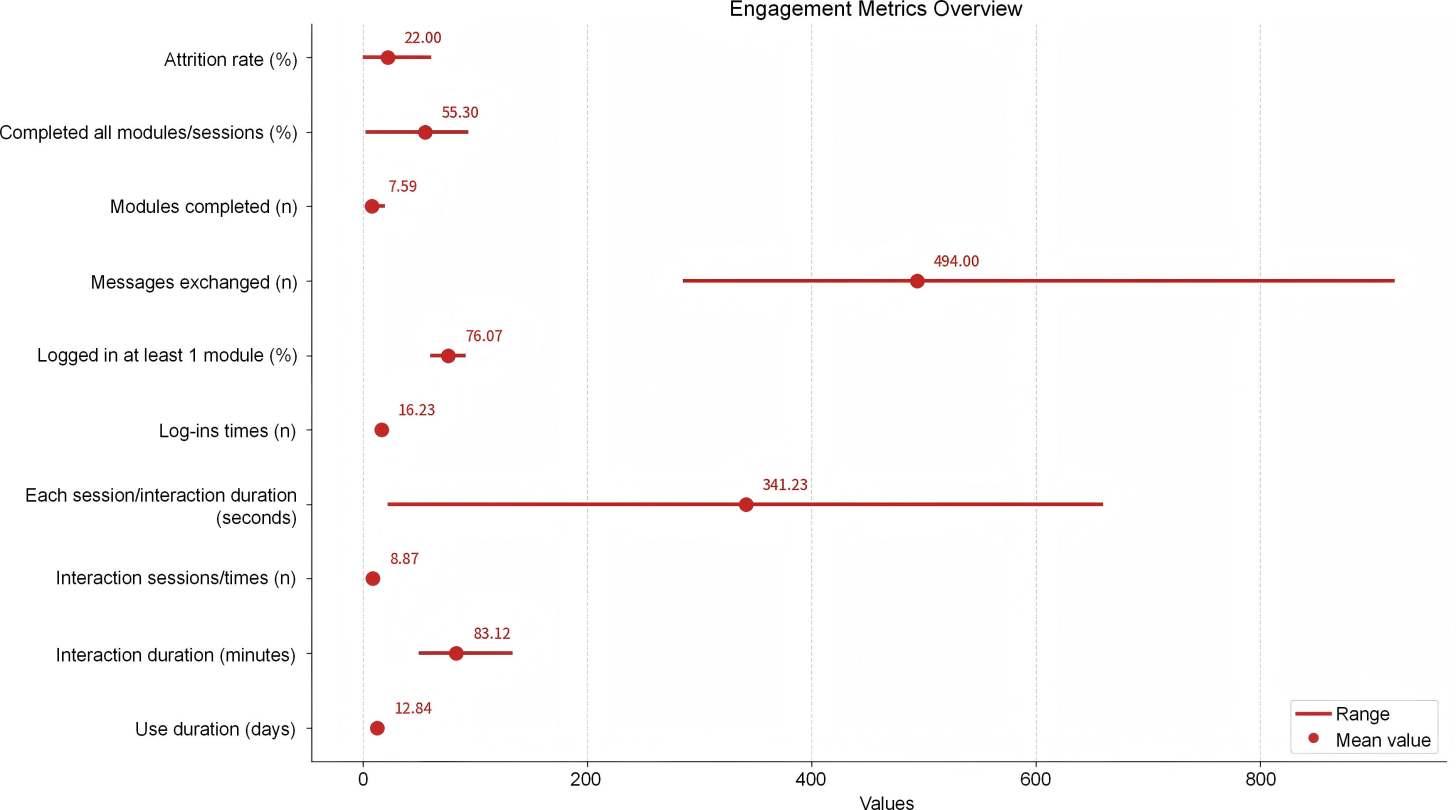
Engagement outcomes for commonly reported metrics. This graph displays the average values for the main reported (at least 2 studies) engagement metrics, calculated from the means reported by the studies.

Engagement outcomes, synthesized from commonly reported metrics (reported by at least 2 studies), revealed distinct interaction patterns (Figure 7). Although the mean attrition was moderate (22%), participants engaged on average for 12.84 days over the 4-week intervention period (range 9‐16 days). Program completion rates varied widely (2.8%‐94%), and reports of interaction duration were inconsistent (50‐134 minutes). Notably, 1 study demonstrated intensive daily interaction (25.54 times per day), while only 10% of studies have reported interaction session metrics (mean sessions: 8.87). In line with these findings, the mean number of messages exchanged was substantial (494 messages per user), and participants completed an average of 7.59 modules.

### Acceptability

Two studies [22,43] assessed overall acceptability using the user version of the Mobile App Rating Scale (total score range 1‐5), reporting an average score of 3.99. One study [38] measured overall acceptability via the Usage Rating Profile—Intervention (total score range 6‐36), reporting a mean score of 29, which indicated high acceptability. User satisfaction with chatbot interventions was assessed in 9 studies (9/21, 42%) [23,25,27,29,32,35,37,38,45], which included overall, content, functional, and design satisfaction. Among these, 5 studies [25,29,35,37,45] rated overall and content satisfaction using a 5-point Likert scale, and the average scores were 3.72 and 3.69, respectively. Two studies [27,38] reported rates of overall satisfaction with chatbots, with 86% and 83% of participants expressing satisfaction, respectively. One study [27] reported that 80% of participants expressed satisfaction with the therapeutic content delivered by chatbots. Two studies [32,38] evaluated satisfaction using the 8-item Client Satisfaction Questionnaire (total score range 6‐36), the average score was 25.9. Four studies [23,28,38,44] assessed participants’ intent to recommend the chatbots to others in the future, and a majority of participants (71%‐88%) demonstrated a positive likelihood of recommendation. Five studies [25,27,29,37,45] reported that participants felt that they had learned something new from the chatbot, 4 studies [25,27,29,37] noted that participants gained information relevant to daily life, 2 studies [27,37] described participants feeling comfortable with the therapeutic process, and 3 studies [25,27,45] reported increased emotional awareness among participants.

Qualitative feedback on user experience was examined in 14 studies. Participants highlighted several positive features of chatbots, including ease of access, empathy and friendliness, encouragement, usefulness and helpfulness of the information provided, convenience, interesting, and reliability. Negative feedback included technical issues (ie, bugs, glitches, lags, system crashes, and inflexible), content repetitiveness, unnatural or impersonal conversation, misunderstood replies, nonintuitive interfaces, not enough interactivity, and the perceptions that the chatbot was annoying or tedious.

## Discussion

### Main Findings

This systematic review and meta-analysis of RCTs provides a comprehensive synthesis of the efficacy, user engagement, and acceptability of CBT-oriented chatbots for adults with depressive and anxiety symptoms. The findings demonstrate that these interventions significantly alleviate both depressive and anxiety symptoms in the short term, yielding a medium effect size for depression and a small effect size for anxiety. Efficacy was largely consistent across various demographic and clinical factors for depression, whereas for anxiety, chatbots showed greater benefits for individuals with comorbid symptoms, in participant samples with a higher proportion of females, and in trials with higher methodological quality. Furthermore, our synthesis revealed high user acceptability and adequate engagement levels, although technical and personalization barriers remain. Overall, these findings support the usefulness of CBT-oriented chatbots as accessible DMHIs. However, to provide a clinically responsible interpretation, these promising results must be explicitly weighed against the substantial heterogeneity, prevalent ROBs, and the resulting low certainty of evidence.

### Efficacy

The meta-analysis provides robust evidence supporting the short-term efficacy of CBT-oriented psychological chatbots in alleviating depressive symptoms among adults. The observed moderate effect is notably larger than the magnitude reported in a recent broad-spectrum review that included a variety of chatbot interventions [52]. This discrepancy likely stems from our refined inclusion criteria, which were restricted to chatbot interventions based on CBT technology. Given that CBT has consistently demonstrated superiority over other psychotherapeutic interventions and is considered the first-line psychosocial treatment of choice for anxiety and depressive disorders [71,72], this specialized focus may explain the enhanced therapeutic impact. The efficacy for anxiety symptoms was relatively small, which is consistent with previous meta-analyses [49,52]. Our finding indicated that structured cognitive-behavioral components, such as cognitive restructuring, psychoeducation, and exposure techniques, can be effectively delivered through digital interfaces to target anxiety-related symptoms [73,74]. When compared with traditional face-to-face or internet-delivered CBT, which typically yields moderate to large effect sizes for depression and anxiety [75-78], the effects of CBT chatbots are slightly more modest. However, considering the low-barrier and highly scalable nature of chatbots, this moderate efficacy remains clinically highly valuable, especially among individuals reluctant to pursue traditional face-to-face therapy [79].

While the 95% CIs confirmed a significant average therapeutic effect for both depression and anxiety across all included trials, the initial 95% PIs—which estimate the distribution of true effects in future or individual clinical contexts—were wide and crossed zero. This critical distinction indicates that although chatbot interventions are effective on average, their actual therapeutic impact in a new, specific setting might range from highly beneficial to negligible. However, once distinct outlier studies were excluded in the sensitivity analyses, the PIs stabilized and no longer crossed zero. Notably, a closer inspection of these outliers revealed striking methodological consistencies: the majority exhibited a high ROB and predominantly used active control conditions rather than passive controls. In meta-analytic research, comparing an intervention with an active control typically yields smaller relative effect sizes, a finding that, when compounded by high risks of bias, likely drove these studies’ extreme statistical deviations [80,81]. This underscores that the observed heterogeneity is heavily influenced by specific study design choices, particularly the selection of comparator groups and methodological limitations, rather than purely clinical variance among target populations. Consistent with GRADE assessment, this implies that the real-world expected effectiveness of CBT chatbots depends profoundly on rigorous clinical implementation and appropriate baseline comparisons.

Specifically, interpreting these overall efficacy findings requires explicit consideration of 3 interconnected methodological constraints. First, the GRADE results indicate that the overall certainty of evidence ranged from low (for short-term outcomes and long-term depression) to very low (for long-term anxiety). This highlights that our confidence in the precise effect estimates is limited, and future rigorous trials are highly likely to influence these conclusions. Second, the substantial heterogeneity underscores that chatbot efficacy is not universally uniform; while average effects are significant, the actual therapeutic impact is highly sensitive to specific study designs and unmeasured clinical contexts. Third, the ubiquitous ROB, predominantly driven by the lack of participant and personnel blinding, is an inescapable challenge in digital psychotherapy research that can potentially inflate self-reported outcomes [82]. However, it is noteworthy that in our subgroup analyses, studies rated as having a low overall ROB actually demonstrated the largest effect sizes for both depression and anxiety. This nuanced finding implies that while performance bias is prevalent, the therapeutic effects of chatbots are unlikely to be merely an artifact of poor methodological quality. Together, these 3 factors—low GRADE certainty, high heterogeneity, and inherent blinding limitations—dictate that CBT-oriented chatbots should currently be recommended as supplementary clinical tools rather than stand-alone replacements for standard care [83,84].

Regarding long-term sustainability, the therapeutic benefits for depression attenuated over time, and no significant long-term differences were observed for anxiety. This fragility of long-term effects is a common challenge in digital interventions studies [85,86]. Anxiety and depressive symptoms often require ongoing reinforcement of adaptive coping skills; without booster sessions or the therapeutic alliance inherent in face-to-face therapy, the initial gains achieved via chatbots may diminish [87].

### Moderators of Efficacy

For depressive symptoms, the therapeutic efficacy remained remarkably consistent across all examined clinical, demographic, and technological factors. This broad consistency suggests that the core cognitive-behavioral mechanisms delivered via chatbots are robust and highly generalizable for depression. Furthermore, multimodel inference indicated that study design characteristics were the primary drivers of the observed heterogeneity, implying that variance in depression outcomes is largely methodological rather than clinical. While our examined moderators accounted for some of this variance, they did not fully explain it. This residual, unmeasured heterogeneity likely stems from several confounding factors (eg, the specific CBT components) that were inconsistently reported across primary studies [88]. Future studies should systematically report these variables to facilitate more granular meta-analytic investigations.

Conversely, the efficacy for anxiety symptoms was significantly influenced by specific clinical and demographic variables. Notably, chatbots were more effective for adults with comorbid symptoms than for those presenting with anxiety or depression only. This suggests that CBT-oriented chatbots may successfully target transdiagnostic mechanisms—such as cognitive appraisals or emotion exposure—that are prevalent in complex clinical presentations, thereby yielding broader symptom relief [89]. Meta-regression revealed that a higher proportion of female participants was associated with a larger absolute effect size for anxiety reduction. This aligned with previous digital health research indicating that women tend to show greater engagement and adherence to self-guided psychological interventions, possibly due to more favorable attitudes toward emotional expression and help-seeking behavior [90,91]. However, given the limited number of studies included in this meta-regression, these findings should be interpreted with caution. Future research with larger samples is needed to confirm the moderating role of gender composition and explore underlying mechanisms more thoroughly. Additionally, multimodel inference identified study design characteristics as the primary drivers of heterogeneity in anxiety outcomes, substantially outperforming patient profiles and technical features. Specifically, trials with a low ROB yielded significantly larger effect sizes. This highlights that variance in anxiety efficacy is fundamentally methodological, underscoring the imperative for rigorous trial designs to accurately evaluate these digital interventions.

### Engagement

Overall, participant engagement with CBT-oriented chatbots demonstrated promising adherence levels. The average attrition rate in the included studies was 22%. Among these studies, 27 out of 29 (93.1%) studies revealed attrition rates lower than 50%, which is the recommended cutoff threshold for effective DMHIs [92]. This indicates a strong user willingness to interact with CBT-oriented psychological chatbots.

However, synthesizing these engagement outcomes was complicated by widespread inconsistencies in how they were defined and measured, as well as in their levels, which is a common challenge in the field of digital interventions [55,93]. Furthermore, instances where attrition rates were exceptionally high, participant dropout was directly linked to chatbots providing inaccurate or irrelevant responses, which hindered the development of user-chatbot rapport [42].

Given that higher usage rates are generally associated with greater treatment outcomes [94], optimizing engagement remains critical. Future studies should aim to systematically define and standardize engagement metrics, while also exploring user-centered design strategies such as personalized reminders, adaptive pacing, and flexible content delivery.

### Acceptability

Overall, CBT-oriented chatbots appeared to be perceived more positively than negatively by users from various backgrounds regarding acceptability. Across the studies that assessed satisfaction, participants generally expressed high levels of satisfaction with the chatbots, including satisfaction of content, functionality, and design. This suggests that participants found the chatbots generally helpful, accessible, and engaging. Study participants appreciated the features such as convenience, empathy, encouragement, and ease of use. Furthermore, many participants indicated that they would recommend chatbot interventions to others, suggesting considerable potential for broader acceptance. These findings are partially aligned with previous systematic reviews reporting that chatbots provided helpful information and were easy to use [95,96].

Nonetheless, several issues identified from participant feedback require attention in future development. Common criticisms included technical problems, content repetitiveness, impersonal interactions, and the lack of natural conversation flow. These issues are critical, as they can significantly undermine user engagement and potentially hinder therapeutic outcomes [96,97]. Therefore, future research should focus on improving the chatbot’s conversational quality, making interactions more human-like, and reducing technical errors to improve acceptability.

### Implications and Recommendations for Future Studies

The integration of these findings provides actionable guidelines for both clinical implementation and future research: (1) Clinical implementation: CBT chatbots should be positioned within stepped care models as highly scalable, first-line triage interventions, particularly for populations with comorbid mild to moderate symptoms or those reluctant to seek face-to-face care. (2) Standardization of metrics: future RCTs must establish and adhere to standardized engagement metrics (eg, explicit definitions of module completion, log-in frequency, and dropout rates) to allow for more precise meta-analytic syntheses of user adherence. (3) Technological advancement: given user frustrations with rigid, rule-based scripts, future chatbot development should carefully integrate large language models (LLMs). Researchers must design studies to evaluate whether the adaptive, human-like conversational flows of LLMs can improve long-term engagement and efficacy, while ensuring that they strictly adhere to evidence-based CBT frameworks without hallucinating clinical advice. (4) Long-term follow-up: future trials should prioritize extended follow-up periods and test the integration of automated booster sessions to sustain therapeutic gains.

### Limitations

#### Limitations of the Evidence

First, according to the GRADE summary of findings, the certainty of evidence for primary outcomes ranged from very low to low. This was predominantly driven by a high RoB concerning the blinding of participants and personnel, which may inflate self-reported symptom improvements. Second, many studies lacked long-term follow-up data, restricting conclusions about the durability of treatment effects. Third, engagement and acceptability outcomes were inconsistently reported, and key indicators such as log-in frequency, dropout rates, or module completion were available in only a subset of studies, limiting the precision of pooled estimates.

#### Limitations of the Review

First, despite our rigorous inclusion criteria, there remained substantial methodological and technological heterogeneity across the included interventions (eg, varying intervention durations and chatbot functionalities), which limits the broader generalizability of our findings to all digital mental health contexts. Second, although subgroup and meta-regression analyses were conducted, the number of studies available for some analyses may have been insufficient to detect small moderator effects with adequate statistical power and should be interpreted with caution. Finally, this review predominantly captures rule-based or narrowly trained AI systems. As the field rapidly evolves, the absence of studies evaluating modern LLM-powered chatbots limits our ability to comment on the next generation of digital therapeutics.

### Conclusions

This systematic review and meta-analysis is innovative in being the first to systematically isolate theoretically grounded, CBT-oriented chatbots from the broader landscape of digital conversational agents. Unlike broader review studies that pool disparate interventions, our targeted multidimensional approach provides a precise, methodology-driven evaluation of a specific digital therapeutic. By doing so, this review brings to the field critical evidence that these tailored interventions yield significant short-term relief for both depressive and anxiety symptoms. Notably, we identified a specific therapeutic advantage for anxiety management among individuals with comorbid profiles via transdiagnostic mechanisms. However, our findings also highlight the fragility of long-term effects and demonstrate that outcome consistency is profoundly dependent on rigorous study designs and appropriate active comparators. In the real world, the implications of these findings are profound: CBT chatbots hold immense potential to democratize mental health care as scalable, low-barrier first-line tools within stepped care models. To fulfill this potential and sustain long-term engagement, future developments must evolve from rigid, rule-based scripts toward adaptive, LLM-driven architectures, provided they strictly adhere to clinical safety and evidence-based CBT frameworks.

## Supplementary material

10.2196/82677 Multimedia Appendix 1 Search strategies, summary of multiarm studies and data extraction or combination strategies, correlation matrix, study and participants characteristics, intervention and chatbot characteristics, GRADE (Grading of Recommendations Assessment, Development, and Evaluation) summary of findings, and multivariable meta-regression.

10.2196/82677 Multimedia Appendix 2 Funnel plots, Galbraith plots, and meta-regression bubble plots.

10.2196/82677 Checklist 1 PRISMA 2020 expanded checklist, PRISMA 2020 for Abstracts Checklist, and PRISMA-S checklist.

## Abbreviations

AI: artificial intelligence
AIC: Akaike information criterion
BIC: Bayesian information criterion
CBT: cognitive behavioral therapy
DMHI: digital mental health intervention
GRADE: Grading of Recommendations Assessment, Development, and Evaluation
LLM: large language model
MeSH: Medical Subject Headings
PI: prediction interval
PRISMA: Preferred Reporting Items for Systematic Reviews and Meta-Analyses
PRISMA-S: Preferred Reporting Items for Systematic Reviews and Meta-Analyses—Search Extension
RCT: randomized controlled trial
ROB: risk of bias
