# Supplementary material for: Efficacy, User Engagement, and Acceptability of Cognitive Behavioral Therapy–Oriented Psychological Chatbots for Adults With Depressive and/or Anxiety Symptoms: Systematic Review and Meta-Analysis of Randomized Controlled Trials
Source: J Med Internet Res. 2026 May 8;28:e82677. doi: 10.2196/82677 (PMC13154727; doi:10.2196/82677)
Supplement: Multimedia Appendix 1 [file jmir-v28-e82677-s001.docx]

**Multimedia Appendix 1**

**Table S1.** Search strategies.

| 1. Pubmed (via NCBI) Last Date Searched: February 9, 2026 | |
| --- | --- |
| #1 | depression OR "depressive disorder" OR anxiety OR "anxiety disorders" OR "dysthymic disorder" OR "mood disorders"[MeSH Terms] |
| #2 | depressive[Title/Abstract] OR melancholia*[Title/Abstract] OR anxiet*[Title/Abstract] OR hypervigilance[Title/Abstract] OR nervousness[Title/Abstract] OR anxious*[Title/Abstract] OR dysthymi*[Title/Abstract] OR emotion*[Title/Abstract] OR mental[Title/Abstract] OR "affective disorder"[Title/Abstract] OR "affective disorders"[Title/Abstract] OR "mood disorder"[Title/Abstract] |
| #3 | #1 OR #2 |
| #4 | chatbot*[Title/Abstract] OR chatterbot*[Title/Abstract] OR bot[Title/Abstract] OR bots[Title/Abstract] OR "conversational agent"[Title/Abstract] OR "conversational agents"[Title/Abstract] OR "conversational system"[Title/Abstract] OR "conversational systems"[Title/Abstract] OR "conversational assistant"[Title/Abstract] OR "conversational assistants"[Title/Abstract] OR "dialog system"[Title/Abstract] OR "dialog systems"[Title/Abstract] OR "dialogue system"[Title/Abstract] OR "dialogue systems"[Title/Abstract] OR "dialogue agent"[Title/Abstract] OR "dialogue agents"[Title/Abstract] OR "assistance technology"[Title/Abstract] OR "assistance technologies"[Title/Abstract] OR "relational agent"[Title/Abstract] OR "relational agents"[Title/Abstract] OR "virtual human"[Title/Abstract] OR "virtual agent"[Title/Abstract] OR "virtual agents"[Title/Abstract] OR "virtual coach"[Title/Abstract] OR "virtual therapy"[Title/Abstract] OR "virtual voice"[Title/Abstract] OR "virtual reality"[Title/Abstract] OR "virtual assistant"[Title/Abstract] OR "virtual assistants"[Title/Abstract] OR "digital agent"[Title/Abstract] OR "digital agents"[Title/Abstract] OR "digital assistant"[Title/Abstract] OR "digital assistants"[Title/Abstract] OR "digital coach"[Title/Abstract] OR "digital human"[Title/Abstract] OR avatar[Title/Abstract] OR "artificial Intelligence"[Title/Abstract] OR chatGPT[Title/Abstract] OR "chat generative pre-trained transformer"[Title/Abstract] OR "automated agent"[Title/Abstract] OR "automated agents"[Title/Abstract] OR "automated message"[Title/Abstract] OR "automated messages"[Title/Abstract] OR "speech assistant"[Title/Abstract] OR "speech assistants"[Title/Abstract] OR "voice agent"[Title/Abstract] OR "voice assistant"[Title/Abstract] OR "voice assistants"[Title/Abstract] |
| #5 | #3 AND #4 |
| #6 | "randomized controlled trial"[Publication Type] |
| #7 | "randomized controlled trial"[Title/Abstract] OR "controlled clinical trial"[Title/Abstract] OR random*[Title/Abstract] OR trial[Title/Abstract] OR groups[Title/Abstract] |
| #8 | #6 OR #7 |
| #9 | #5 AND #8 |
| 2. Embase (via Elsevier) Last Date Searched: February 9, 2026 | |
| #1 | 'depression'/exp OR 'depression' OR 'anxiety'/exp OR 'anxiety' OR 'anxiety disorder'/exp OR 'anxiety disorder' OR 'dysthymia'/exp OR 'dysthymia' OR 'mood disorder'/exp OR 'mood disorder' |
| #2 | depressive:ab,ti OR melancholia*:ab,ti OR anxiet*:ab,ti OR hypervigilance:ab,ti OR nervousness:ab,ti OR anxious*:ab,ti OR dysthymi*:ab,ti OR emotion*:ab,ti OR mental:ab,ti OR 'affective disorder':ab,ti OR 'affective disorders':ab,ti OR 'mood disorders':ab,ti |
| #3 | #1 OR #2 |
| #4 | 'chatbot'/exp |
| #5 | chatbots:ab,ti OR chatterbot*:ab,ti OR bot:ab,ti OR bots:ab,ti OR 'conversational agent':ab,ti OR 'conversational agents':ab,ti OR 'conversational system':ab,ti OR 'conversational systems':ab,ti OR 'conversational assistant':ab,ti OR 'conversational assistants':ab,ti OR 'dialog system':ab,ti OR 'dialog systems':ab,ti OR 'dialogue system':ab,ti OR 'dialogue systems':ab,ti OR 'dialogue agent':ab,ti OR 'dialogue agents':ab,ti OR 'assistance technology':ab,ti OR 'assistance technologies':ab,ti OR 'relational agent':ab,ti OR 'relational agents':ab,ti OR 'virtual human':ab,ti OR 'virtual agent':ab,ti OR 'virtual agents':ab,ti OR 'virtual coach':ab,ti OR 'virtual therapy':ab,ti OR 'virtual voice':ab,ti OR 'virtual reality':ab,ti OR 'virtual assistant':ab,ti OR 'virtual assistants':ab,ti OR 'digital agent':ab,ti OR 'digital agents':ab,ti OR 'digital assistant':ab,ti OR 'digital assistants':ab,ti OR 'digital coach':ab,ti OR 'digital human':ab,ti OR avatar:ab,ti OR 'artificial intelligence':ab,ti OR chatgpt:ab,ti OR 'chat generative pre-trained transformer':ab,ti OR 'automated agent':ab,ti OR 'automated agents':ab,ti OR 'automated message':ab,ti OR 'automated messages':ab,ti OR 'speech assistant':ab,ti OR 'speech assistants':ab,ti OR 'voice agent':ab,ti OR 'voice assistant':ab,ti OR 'voice assistants':ab,ti |
| #6 | #4 OR #5 |
| #7 | 'randomized controlled trial':ab,ti OR 'controlled clinical trial':ab,ti OR random*:ab,ti OR trial:ab,ti OR 'groups':ab,ti |
| #8 | #3 AND #6 AND #7 |
| 3. Web of Science (via Clarivate) Last Date Searched: February 9, 2026 | |
| #1 | TS=(depressi* OR melancholia* OR anxiet* OR hypervigilance OR nervousness OR anxious* OR dysthymi* OR emotion* OR mental OR "affective disorder" OR "affective disorders" OR "mood disorder" OR "mood disorders") |
| #2 | TS=(chatbot* OR chatterbot* OR bot OR bots OR “conversational agent” OR “conversational agents” OR “conversational system” OR “conversational systems” OR “conversational assistant” OR “conversational assistants” OR “dialog system” OR “dialog systems” OR “dialogue system” OR “dialogue systems” OR "dialogue agent" OR "dialogue agents" OR “assistance technology” OR “assistance technologies” OR “relational agent” OR “relational agents” OR “virtual human” OR “virtual agent” OR “virtual agents” OR “virtual coach” OR “virtual therapy” OR "virtual voice" OR “virtual reality” OR “virtual assistant” OR “virtual assistants” OR “digital agent” OR “digital agents” OR “digital assistant” OR “digital assistants” OR “digital coach” OR “digital human” OR avatar OR “artificial Intelligence” OR chatGPT OR “chat generative pre-trained transformer” OR “automated agent” OR “automated agents” OR “automated message” OR “automated messages” OR “speech assistant” OR “speech assistants” OR “voice agent” OR “voice assistant” OR “voice assistants”) |
| #3 | TS=("randomized controlled trial" OR "controlled clinical trial" OR random* OR trial OR groups) |
| #4 | #1 AND #2 AND #3 |
| 4. Cochrane Central Register of Controlled Trials (via Wiley) Last Date Searched: February 9, 2026 | |
| #1 | MeSH descriptor: [Depression] explode all trees |
| #2 | MeSH descriptor: [Depressive Disorder] explode all trees |
| #3 | MeSH descriptor: [Anxiety] explode all trees |
| #4 | MeSH descriptor: [Anxiety Disorder] explode all trees |
| #5 | MeSH descriptor: [Dysthymic Disorder] explode all trees |
| #6 | MeSH descriptor: [Mood Disorders] explode all trees |
| #7 | #1 OR #2 OR #3 OR #4 OR #5 OR #6 |
| #8 | (depressive OR melancholia* OR anxiet* OR hypervigilance OR nervousness OR anxious* OR dysthymi* OR emotion* OR mental OR "affective disorder" OR "affective disorders" OR "mood disorder"):ti,ab,kw |
| #9 | #7 OR #8 |
| #10 | (chatbot* OR chatterbot* OR bot OR bots OR “conversational agent” OR “conversational agents” OR “conversational system” OR “conversational systems” OR “conversational assistant” OR “conversational assistants” OR “dialog system” OR “dialog systems” OR “dialogue system” OR “dialogue systems” OR "dialogue agent" OR "dialogue agents" OR “assistance technology” OR “assistance technologies” OR “relational agent” OR “relational agents” OR “virtual human” OR “virtual agent” OR “virtual agents” OR “virtual coach” OR “virtual therapy” OR "virtual voice" OR “virtual reality” OR “virtual assistant” OR “virtual assistants” OR “digital agent” OR “digital agents” OR “digital assistant” OR “digital assistants” OR “digital coach” OR “digital human” OR avatar OR “artificial Intelligence” OR chatGPT OR “chat generative pre-trained transformer” OR “automated agent” OR “automated agents” OR “automated message” OR “automated messages” OR “speech assistant” OR “speech assistants” OR “voice agent” OR “voice assistant” OR “voice assistants”):ti,ab,kw |
| #11 | #9 AND #10 |
| 5. CINAHL (via EBSCOhost) Last Date Searched: February 9, 2026 | |
| S1 | MH(depression OR "depressive disorder" OR anxiety OR "anxiety disorders" OR "dysthymic disorder" OR "mood disorders") |
| S2 | TI(depressive OR melancholia* OR anxiet* OR hypervigilance OR nervousness OR anxious* OR dysthymi* OR emotion* OR mental OR "affective disorder" OR "affective disorders" OR "mood disorder") or AB(depressive OR melancholia* OR anxiet* OR hypervigilance OR nervousness OR anxious* OR dysthymi* OR emotion* OR mental OR "affective disorder" OR "affective disorders" OR "mood disorder") |
| S3 | S1 OR S2 |
| S4 | TI(chatbot* OR chatterbot* OR bot OR bots OR “conversational agent” OR “conversational agents” OR “conversational system” OR “conversational systems” OR “conversational assistant” OR “conversational assistants” OR “dialog system” OR “dialog systems” OR “dialogue system” OR “dialogue systems” OR "dialogue agent" OR "dialogue agents" OR “assistance technology” OR “assistance technologies” OR “relational agent” OR “relational agents” OR “virtual human” OR “virtual agent” OR “virtual agents” OR “virtual coach” OR “virtual therapy” OR "virtual voice" OR “virtual reality” OR “virtual assistant” OR “virtual assistants” OR “digital agent” OR “digital agents” OR “digital assistant” OR “digital assistants” OR “digital coach” OR “digital human” OR avatar OR “artificial Intelligence” OR chatGPT OR “chat generative pre-trained transformer” OR “automated agent” OR “automated agents” OR “automated message” OR “automated messages” OR “speech assistant” OR “speech assistants” OR “voice agent” OR “voice assistant” OR “voice assistants”) OR AB (chatbot* OR chatterbot* OR bot OR bots OR “conversational agent” OR “conversational agents” OR “conversational system” OR “conversational systems” OR “conversational assistant” OR “conversational assistants” OR “dialog system” OR “dialog systems” OR “dialogue system” OR “dialogue systems” OR "dialogue agent" OR "dialogue agents" OR “assistance technology” OR “assistance technologies” OR “relational agent” OR “relational agents” OR “virtual human” OR “virtual agent” OR “virtual agents” OR “virtual coach” OR “virtual therapy” OR "virtual voice" OR “virtual reality” OR “virtual assistant” OR “virtual assistants” OR “digital agent” OR “digital agents” OR “digital assistant” OR “digital assistants” OR “digital coach” OR “digital human” OR avatar OR “artificial Intelligence” OR chatGPT OR “chat generative pre-trained transformer” OR “automated agent” OR “automated agents” OR “automated message” OR “automated messages” OR “speech assistant” OR “speech assistants” OR “voice agent” OR “voice assistant” OR “voice assistants”) |
| S5 | TI('randomized controlled trial' OR 'controlled clinical trial' OR random* OR trial OR groups) OR AB('randomized controlled trial' OR 'controlled clinical trial' OR random* OR trial OR groups) |
| S6 | S3 AND S4 AND S5 |
| 6. PsycINFO (via APA PsycNet) Last Date Searched: February 9, 2026 | |
| S1 | mainsubject(depression OR "depressive disorder" OR anxiety OR "anxiety disorders" OR "dysthymic disorder" OR "mood disorders") |
| S2 | tiab(depressive OR melancholia* OR anxiet* OR hypervigilance OR nervousness OR anxious* OR dysthymi* OR emotion* OR mental OR "affective disorder" OR "affective disorders" OR "mood disorder") |
| S3 | [S1] OR [S2] |
| S4 | tiab(chatbot* OR chatterbot* OR bot OR bots OR “conversational agent” OR“conversational agents” OR “conversational system” OR “conversational systems” OR “conversational assistant” OR “conversational assistants” OR “dialog system” OR “dialog systems” OR “dialogue system” OR “dialogue systems” OR "dialogue agent" OR "dialogue agents" OR “assistance technology” OR “assistance technologies” OR “relational agent” OR “relational agents” OR “virtual human” OR “virtual agent” OR “virtual agents” OR “virtual coach” OR “virtual therapy” OR "virtual voice" OR “virtual reality” OR “virtual assistant” OR “virtual assistants” OR “digital agent” OR “digital agents” OR “digital assistant” OR “digital assistants” OR “digital coach” OR “digital human” OR avatar OR “artificial Intelligence” OR chatGPT OR “chat generative pre-trained transformer” OR “automated agent” OR “automated agents” OR “automated message” OR “automated messages” OR “speech assistant” OR “speech assistants” OR “voice agent” OR “voice assistant” OR “voice assistants”) |
| S5 | [S3] AND [S4] |
| S6 | tiab("randomized controlled trial" OR "controlled clinical trial" OR random* OR trial OR groups) |
| S7 | [S5] AND [S6] |
| 1. CNKI (Chinese) Last Date Searched: February 9, 2026 | |
| 1 | (TKA='抑郁' OR TKA='焦虑' OR TKA='负性情绪' OR TKA='负面情绪') AND (TKA='数字化' OR TKA='聊天机器人' OR TKA='人工智能' OR TKA='对话' OR TKA='虚拟' OR TKA='语音助手' OR TKA='VR' OR TKA='AI' OR TKA='ChatGPT') |
| 1. WanFang (Chinese) Last Date Searched: February 9, 2026 | |
| 1 | 题名或关键词:(抑郁 or 焦虑 or 负性情绪 or 负面情绪) and 题名或关键词:(数字化 or 聊天机器人 or 人工智能 or 对话 or 虚拟 or 语音助手 or VR or AI or ChatGPT) |
| 1. VIP Databases (Chinese) Last Date Searched: February 9, 2026 | |
| 1 | M=(抑郁 OR 焦虑 OR 负性情绪 OR 负面情绪) AND M=(数字化 OR 聊天机器人 OR 人工智能 OR 对话 OR 虚拟 OR 语音助手 OR VR OR AI OR ChatGPT) |

**Table S2.** Summary of multi-arm studies and data extraction/combination strategies.

| **Study** | **Study Design (Original Arms)** | **Arms Extracted / Combined for Meta-analysis** | **Decision and Justification** |
| --- | --- | --- | --- |
| **Fulmer et al (2018) [27]** | 3-arm RCT Group 1: Tess (2 weeks) Group 2: Tess (4 weeks)  Group 3: Information-only control group | **Combined (Group 1 + Group 2)** vs. Group 3 | Both active intervention arms involved CBT-oriented chatbots but with different intervention durations. They were combined into a single composite group to assess overall efficacy. |
| **Danieli et al (2022) [35]** | 4-arm RCT Group 1: SMT-CBT Group 2: SMT-CBT & PHA Group 3: PHA-only Group 4: Test-only | **Group 3** vs. Group 4 | PHA is a mobile personal health care agent with conversational AI. Only Group 3 as active intervention arm was extracted. |
| **He et al (2022) [25]** | 3-arm RCT Group 1: XiaoE Group 2: e-book  Group 3: Xiaoai | **Group 1** vs. Group 2 | XiaoE is an unguided CBT-based chatbot. Xiaoai is a chatbot in China designed to cater to the demands of a wider audience for small talk. The Xiaoai group was excluded. |
| **Sabour et al (2023) [23]** | 3-arm RCT Group 1: Emohaa (CBT chatbot)  Group 2: ES-Bot Group 3: Waitlist control | **Group 1** vs. Group 3 | Only the Emohaa chatbot arm was extracted. The ES-Bot group was excluded as it did not involve CBT technology. |
| **Chan et al (2024) [20]** | 5-arm RCT Group 1: dCBTi-therapist Group 2: dCBTi-assistant Group 3: dCBTi-chatbot Group 4: dCBTi-unguided Group 5: dSH (digital sleep hygiene and self-monitoring control) | **Group 3** vs. Group 5 | dCBTi-therapist: digital cognitive behavioral therapy for insomnia with chatbot-based coaching and therapist support. dCBTi-assistant: digital cognitive behavioral therapy for insomnia with chatbot-based coaching and research assistant support. dCBTi-chatbot: digital cognitive behavioral therapy for insomnia with chatbot-based coaching only. dCBTi-unguided: digital cognitive behavioral therapy for insomnia without any coaching.  Only the dCBTi-chatbot group was extracted to isolate the efficacy of chatbot-driven intervention. The other active intervention arms were excluded. |

**Table S3.** Correlation matrix of predefined moderators for depressive and anxiety symptoms.

| **Variables** | **Age** | **Female proportion** | **Participants** | **Control Type** | **Intervention** duration | **Platform** | **Chatbot type** | **Country** | **ROB** |
| --- | --- | --- | --- | --- | --- | --- | --- | --- | --- |
| **Age** | 1 | -0.0138 | 0.2887 | 0.3018 | 0.1802 | **0.4298*** | 0.2624 | -0.3358 | -0.0967 |
| **Female proportion** | 0.0749 | 1 | 0.3216 | 0.3084 | 0.2051 | -0.2082 | **-0.5381*** | -0.0537 | -0.131 |
| **Participants** | 0.3086 | 0.3756 | 1 | 0.1288 | 0.2642 | 0.3024 | -0.303 | 0.0543 | -0.2716 |
| **Control Type** | 0.3086 | 0.3859 | 0.2262 | 1 | 0.0367 | 0.1241 | -0.1288 | -0.3024 | 0.044 |
| **Intervention duration** | 0.0889 | 0.2163 | 0.2297 | 0.0345 | 1 | -0.0526 | 0.044 | -0.0301 | 0.1722 |
| **Platform** | **0.4427*** | -0.1529 | 0.2562 | 0.0976 | 0.0235 | 1 | 0.0543 | -0.2698 | 0.1277 |
| **Chatbot type** | 0.1749 | **-0.5660*** | -0.3810 | -0.2262 | 0.0345 | 0.0610 | 1 | -0.2326 | -0.1908 |
| **Country** | -0.3689 | -0.1160 | 0.0610 | -0.2562 | -0.0942 | -0.3500 | -0.2196 | 1 | -0.2104 |
| **ROB** | -0.0743 | 0.0353 | -0.1354 | 0.1354 | 0.3002 | 0.1619 | -0.2370 | -0.2370 | 1 |

ABBREVIATIONS: ROB = risk of bias

Notes: Values represent Spearman's rank correlation coefficients (r). The lower-left triangle (pink background) displays the correlations within the depressive symptoms dataset, while the upper-right triangle (blue background) displays the correlations within the anxiety symptoms dataset.

* Indicates statistical significance at *p* < 0.05.

**Table S4.** Study and participants characteristics.

| **Author (year)** | **Country** | **Design** | **Nature of Participant** | **Sample Size** | **Mean Age (SD)** | **Female (%)** | **Intervention group** | **Control group** | **Depression/Anxiety Measures** | **ITT Data** |
| --- | --- | --- | --- | --- | --- | --- | --- | --- | --- | --- |
| McFadyen et al (2026) [29] | US | 2-arm RCT | Adults with elevated self-reported symptoms of anxiety or depression | I:322 C:209 | 36.97 (11.64) | 375 (71%) | Limbic Care app | Digital workbook | PHQ-9 /GAD-7 | Yes^#^ |
| Bryant et al (2026) [17] | Jordan | 2-arm RCT | Young adults with moderate/high psychological distress | I:171 C:173 | 19.7 (1.1) | 252 (73%) | STARS intervention | Enhanced Care as Usual | HSCL-25-D  /HSCL-25-A | Yes |
| Allen et al (2026) [18] | UK | 2-arm RCT | Adults with generalized anxiety symptoms | I:147 C:169 | 37.75 (10.66) | 143 (61%) | AI-powered mental health app | NHS self-help website | PHQ-9 /GAD-7 | Yes |
| Wang et al (2025) [19] | China | 2-arm RCT | University students with depressive or anxiety symptoms | I:50 C:50 | 20.8 (2.2) | 62 (62%) | CBT-based AI chatbot | Waitlist | CED-D-10 /GAD-7 | Yes |
| Tong et al (2025) [30] | China | 2-arm RCT | Adults with depressive or anxiety symptoms | I:140 C:145 | 26.45 (8.37) | 216 (76%) | Chatbot group | Waitlist | PHQ-9 /GAD-7 | Yes |
| Chiu et al (2025) [31] | China | 2-arm RCT | Adults with insomnia symptoms had comorbid depression and anxiety | I:35 C:31 | NR | 57 (86%) | CBT-I chatbot | Static sleep education website | PHQ-9, BDI /BAI | No |
| de Graaff et al (2025) [32] | Jordan | 2-arm RCT | Young people with self-reported elevated levels of psychological distress | I:30 C:30 | 19.68 (1.16) | 49 (82%) | STARS intervention | Enhanced Care as Usual | HSCL-25-D  /HSCL-25-A | No |
| Ulrich et al (2024) [22] | Switzerland | 2-arm RCT | College students experiencing stress with depressive or anxiety symptoms | I:70 C:70 | 26.71 (6.29) | 103 (73.6%) | MISHA app intervention | Waitlist | PHQ-9  /GAD-7 | No |
| MacNeill et al (2024) [33] | Canada | 2-arm RCT | Arthritis or diabetes adults with depressive or anxiety symptoms | I:34 C:34 | 42.87 (11.27) | 47 (69%) | Mental health chatbot app | No intervention | PHQ-9  /GAD-7 | No |
| Chan et al (2024) [20] | China | 5-arm RCT | Insomnia adults with depressive or anxiety symptoms | I:26 C:30 | 35.18 (11.94) | 48 (85.7%) | dCBTi-chatbot | Digital sleep hygiene and self-monitoring | PHQ-9  /GAD-7 | Yes |
| Karkosz et al (2024) [34] | Poland | 2-arm RCT | Adults with subclinical depression or anxiety | I:40  C:41 | 25.67 (4.59) | 58 (71.6%) | Fully automated  chatbot | Self-help book | PHQ-9  /STAI | No |
| Vereschagin et al (2024) [21] | Canada | 2-arm RCT | College students with depressive or anxiety symptoms | I:743  C:746 | 20^*^ | 1045 (70.3%) | Minder mobile app | Waitlist | PHQ-9  /GAD-7 | No |
| Ulrich et al (2024) [43] | Switzerland Germany  Austria | 2-arm RCT | Frequent headaches adults with depressive or anxiety symptoms | I:110  C:88 | 38.7 (12.14) | 172 (86.9%) | Conversational agent intervention | Waitlist | PHQ-9  /GAD-7 | No |
| Sabour et al (2023) [23] | China | 3-arm RCT | Adults with depressive or anxiety symptoms | I:72  C:105 | 31.65 (7.73) | 138 (78%) | CBT-Bot | Blank control | PHQ-9  /GAD-7 | Yes |
| Kannampallil et al (2023) [24] | US | 2-arm RCT | Adults with mild-to-moderate depression and/or anxiety | I:42  C:21 | 37.8 (12.4) | 43 (68.3%) | Lumen intervention | Waitlist | HADS-D  /HADS-A | No |
| He et al (2022) [25] | China | 3-arm RCT | College students with depressive symptoms | I:49  C:49 | 18.86 (0.87) | 36 (36.7%) | CBT-based mental health chatbot | E-book | PHQ-9 | Yes |
| Liu et al (2022) [26] | China | 2-arm RCT | College students with depression | I:41  C:42 | 23.08 (1.76) | 46 (55.4%) | Chatbot-delivered intervention | Bibliotherapy | PHQ-9  /GAD-7 | Yes |
| Danieli et al (2022) [35] | Italy | 4-arm RCT | Participants with stress  symptoms and mild-to-moderate anxiety | I:8  C:10 | 56.5 (6.67) | 14 (77.8%) | Mobile personal health care agent | Waitlist | PHQ-8  /GAD-7 | Yes |
| Fitzsimmons-Craft et al (2022) [41] | US | 2-arm RCT | High ED risk in females with depressive or anxiety symptoms | I:352  C:348 | 21.08 (3.09) | 700 (100%) | Chatbot based on the StudentBodies© program | Waitlist | PHQ-8  /GAD-7 | No |
| Klos et al (2021) [42] | Argentina | 2-arm RCT | College students with depressive or anxiety symptoms | I:99 C:82 | NR | 158 (87.2%) | AI-based chatbot | E-book | PHQ-9  /GAD-7 | No |
| Hunt et al (2021) [36] | US | 2-arm RCT | IBS with depressive symptoms | I:62  C:59 | 32 (10.2) | 91 (75.2%) | Zemedy app intervention | Waitlist | PHQ-9  /DASS-A | No |
| Jang et al (2021) [37] | Korea | 2-arm RCT | Attention deficit individuals with depressive symptoms | I:23 C:23 | 25.1 (7.5) | 26 (56.5%) | Chatbot app intervention | Paperback | QIDS-SR  /SAS | Yes |
| Prochaska et al (2021) [38] | US | 2-arm RCT | Substance abuse disorder adults with depressive or anxiety symptoms | I:88  C:92 | 40 (12) | 117 (65%) | W-SUDs | Waitlist | PHQ-8  /GAD-7 | No |
| Oh et al (2020) [39] | Korea | 2-arm RCT | Mild-to-severe panic symptoms patients with depressive or anxiety symptoms | I:21  C:20 | 41.45 (11.69) | 21 (51.2%) | Mobile app-based interactive CBT using the chatbot | Paperback | HADS-D  /HADS-A | Yes |
| Greer et al (2019) [44] | US | 2-arm RCT | Young adults within 5 years of completing active cancer treatment with moderate anxiety and mild depression | I:25  C:20 | 25 (2.9) | 36 (80%) | Vivibot app intervention | Waitlist | PROMIS-D  /PROMIS-A | No |
| Fulmer et al (2018) [27] | US | 3-arm RCT | College students with depressive and anxiety symptoms | I:50 (24/26)  C:24 | 22.90 (4.38) | 52 (69%) | AI-based chatbot intervention | E-book | PHQ-9  /GAD-7 | No |
| Fitzpatrick et al (2017) [45] | US | 2-arm RCT | College students with depressive and anxiety symptoms | I:34  C:36 | 22.2 (2.3) | 47 (67%) | Woebot app intervention | E-book | PHQ-9  /GAD-7 | No |
| Pinto et al (2016) [40] | US | 2-arm RCT | Young adults with depressive symptoms | I:12  C:16 | 22 (2.5) | 19 (67%) | Avatar-based mental health intervention | Screen-based health  education | HADS-D | No |
| Burton et al (2016) [28] | Romania Spain and Scotland  UK | 2-arm RCT | Patients with MDD and current mild/moderate depressive symptoms | I:13  C:14 | 38.77 (11.47) | 18 (66.7%) | AI-embodied interactive  system + TAU | TAU | BDI-2 | Yes |

^*^ median, ^#^ modified Intention to Treat

ABBREVIATIONS: SD = standard deviation; RCT = randomised controlled trial; I = intervention group; C = control group; Hopkins Symptom Checklist-25 = HSCL-25; PHQ-9 = Patient Health Questionnaire 9-item scale; STAI = State-Trait Anxiety Inventory; PANAS = Positive and Negative Affect Schedule; PSWQ = Penn State Worry Questionnaire; SWLS = Satisfaction with Life Scale; R-UCLA = Revised UCLA Loneliness Scale; GAD-7 = Generalized Anxiety Disorder 7-item scale; SWEMWS = Short Warwick-Edinburgh Mental Wellbeing Scale; PSS-10 = Perceived Stress Scale-10 items; HMSE-G-SF = Headache management self-efficacy; SPSI-R:S = Social Problem-solving Index-Revised Short Form; PPO = positive problem orientation; NPO = negative problem orientation; RPS = rational problem-solving style; ICS = impulsive/careless problem-solving style; AS = avoidant problem-solving style; DAS = Dysfunctional Attitudes Scale; MHSES = Mental Health Self-Efficacy Scale; PHQ-8 = Patient Health Questionnaire 8-item scale; PSS-10 = Perceived Stress Scale; OSI = Occupational Stress Inventory; ED = eating disorder; WCS = Weight concerns scale; SATAQ-4R, Sociocultural Attitudes Toward Appearance Questinnaire-4R; EDE-Q = Eating Disorder Examination-Questionnaire; DASS-A = Depression Anxiety Stress Scale - Anxiety; DASS-S = Depression Anxiety Stress Scale - Stress; IBS-QOL = IBS-Quality of Life; QIDS-SR = Quick Inventory of Depressive Symptoms-Self-Report; SAS = Self-rating Anxiety Scale; BSCQ = Brief Situational Confidence Questionnaire; HADS-D = Hospital Anxiety and Depression Scale - Depression; HADS-A = Hospital Anxiety and Depression Scale - Anxiety; PDSS = Panic Disorder Severity Scale; APPQ = Albany Panic and Phobia Questionnaire; BSQ = Body Sensations Questionnaire (BSQ); ACQ = Anxiety Control Questionnaire (ACQ); PROMIS-D = Patient-Reported Outcomes Measurement Information System - Depression; PROMIS-A = Patient-Reported Outcomes Measurement Information System - Anxiety; BIPQ = Brief Illness Perception Questionnaire; TAU = treatment as usual; BDI-2 = Beck Depression Inventory II; MDD = major depressive disorder; DAS-SF = Dysfunctional Attitudes Scale-Short Form; EQ-5D-5L = EuroQol 5D.

**Table S5.** Intervention and chatbot characteristics.

| **Author (year)** | **CBT Component** | **Intervention Duration** | **No. of Sessions** | **Duration of Each Session** | **Follow-up**  **Length** | **Chatbot Name** | **Platform** | **Chatbot Type** | **Dialogue Initiative** | **Interaction Frequency** | **Input** | **Output** |
| --- | --- | --- | --- | --- | --- | --- | --- | --- | --- | --- | --- | --- |
| McFadyen et al (2026) [29] | Psychoeducation Thought records Behavioral activation Mindfulness practices | 6 weeks | NR | NR | None | Limbic Care | Stand-alone mobile app | AI-based (LLM, ML) | Both | 4 times per week | Text | Text Image |
| Bryant et al (2026) [17] | Psychoeducation Controlled breathing Grounding Identifying master, pleasure, and/or socialconnection Problem management Adaptive self‑talk, relapse prevention | 5 weeks | 10 | 10 - 25 minutes | 3 months | SALAM | Multimodal (via Website) | Rule‑based | Chatbot | 10 lessons completed over 5 weeks | Text | Text  Audio Video |
| Allen et al (2026) [18] | Exploration of feelings, behaviours, and emotions Interactive evidence-based exercises (e.g., breathing exercises, meditation) | 2 weeks | NR | 5-15 minutes | 8 weeks and 12 weeks | PATH | Stand-alone mobile app | Rule-based | Both | At least 5 times per week | Text Voice | Text |
| Wang et al (2025) [19] | Cognitive restructuring Behavioral activation Problem-solving training Mindfulness exercises Social skills development | 1 week | 7 | 20-30 minutes | None | Psy-Bot | Stand-alone mobile app | AI-based (NLP) | Both | Daily | Text | Text Images Audio |
| Tong et al (2025) [30] | Behavioral activation Psychoeducation Mindfulness, and muscle relaxation practices | 10 days | 10 | 10-15 minutes | 1 month | Boon | Multimodal (via Website) | Rule‑based | Both | Daily | Text | Text |
| Chiu et al (2025) [31] | Dysfunctional belief management Behavioral activation (sleep restriction, stimulus control) Relaxation techniques Psychoeducation Self-monitoring (sleep/mood diaries) | 4 weeks | 7 | NR | None | NR | Multimodal (via LINE) | Rule‑based | Both | Daily | Text | Text  Video Emoji |
| de Graaff et al (2025) [32] | Psychoeducation Emotion regulation Behavioral activation Problem management  Thought challenging  Relapse prevention | 8 weeks | 10 | 20 minutes | None | Salam | Multimodal (via Website) | Rule-based | Chatbot | Twice per week | Text | Text  Audio Video |
| Ulrich et al (2024) [22] | Cognitive restructuring,  identification, evaluation Modification of maladaptive thought patterns  Behavioral activation Activity monitoring | 24 to 54 days | 12 | NR | None | MISHA | Stand-alone mobile app | Rule-based | Chatbot | Once every 2 to 4 days | Text  Emoji | Text  Audio Video Emoji |
| MacNeill et al (2024) [33] | NR | 4 weeks | NR | NR | None | Wysa | Stand-alone mobile app | AI-based  (NLP) | Chatbot | At least twice per week | Text | Text |
| Chan et al (2024) [20] | Sleep restriction Stimulus control Sleep hygiene Psychoeducation Relaxation Cognitive therapy | 6 weeks | 6 | NR | 4 weeks | Sleep Sensei | Stand-alone mobile app | Rule-based | Chatbot | Daily access | Text | Text  Video |
| Karkosz et al (2024) [34] | Cognitive biases recognition Psychoeducation ABC technique Gratitude practice | 2 weeks | NR | NR | 1 month | Fido | Multimodal (via Facebook message) | AI-based  (ML) | Both | Freely access | Text | Text  Emoji |
| Vereschagin et al (2024) [21] | NR | 30 days | NR | NR | None | Minder | Stand-alone mobile app | Rule-based | Both | Freely access | Text | Text  Audio Video |
| Ulrich et al (2024) [43] | Psychoeducation Reflection of behavior Behavioral intention Action planning Relaxation and imagination | 24 to 60 days | 7 | NR | None | BalanceUP | Stand-alone mobile app | Rule-based | Chatbot | Freely access | Text  Emoji | Text  Emoji Image Video |
| Sabour et al (2023) [23] | Automatic thoughts training Guided expressive writing | 3 weeks | NR | NR | 1 month | Emohaa | Multimodal (via WeChat) | Rule-based | Chatbot | Daily access | Text | Text |
| Kannampallil et al (2023) [24] | Problem-solving therapy | 16 weeks | 8 | 12 minutes | None | Lumen | Multimodal (via Amazon's Alexa) | AI-based  (ML, NLP) | Chatbot | 4 weekly and the following 4 biweekly | Voice | Voice  Text |
| He et al (2022) [25] | Psychoeducation Cognitive distortions Improve self-esteem Mindfulness meditation Mental energy  Natural connection  Self-help  Escape from loneliness | 1 week | 7 | NR | 1 month | XiaoE | Multimodal (via WeChat) | AI-based  (NLP, DL) | Chatbot | Daily access | Text | Text  Image Voice |
| Liu et al (2022) [26] | NR | 16 weeks | NR | NR | None | XiaoNan | Multimodal (via WeChat) | AI-based  (NLP, ML) | Both | Freely access | Text Voice | Text |
| Danieli et al (2022) [35] | ABC technique | 8 weeks | NR | NR | 3 months | TEO | Stand-alone mobile app | AI-based  (NLP) | Both | NR | Text | Text |
| Fitzsimmons-Craft et al (2021) [41] | NR | 4 weeks | 8 | 10 minutes | 3 months  6 months | Tessa | Multimodal (via SMS or Facebook message) | Rule-based | Chatbot | Twice a week | Text  Emoji | Text  Emoji Infographic |
| Klos et al (2021) [42] | NR | 8 weeks | NR | NR | None | Tess | Multimodal platform (via Facebook message) | AI-based  (NLP) | Chatbot | Once a day during the initial weeks and every other day in the following weeks | Text | Text Emojis |
| Hunt et al (2021) [36] | Psychoeducation Relaxation training  Exercise Cognitive restructuring  and decatastrophizing Exposure exercises to reduce avoidance Behavioral experiments | 8 weeks | 10 | NR | 3 months | Zemedy | Stand-alone mobile app | Rule-based | Chatbot | Daily access | Text | Text |
| Jang et al (2021) [37] | Psychoeducation Behavior change strategies Emotional control techniques Mindfulness training | 4 weeks | 40 | NR | None | Todaki | Stand-alone mobile app | Rule-based | Both | Daily access | Text | Text  Emoji Image |
| Prochaska et al (2021) [38] | Mood tracking Behavioral pattern insight | 8 weeks | 66 | NR | None | Woebot | Stand-alone mobile app | AI-based (NLP) | Chatbot | Daily access | Text | Text  Emoji Image |
| Oh et al (2020) [39] | Address distorted thoughts Interoceptive exposure training  In vivo exposure session guidance | 4 weeks | 20 | NR | None | Todaki | Stand-alone mobile app | Rule-based | Both | Freely access | Text | Text  Audio Image Voice |
| Greer et al (2019) [44] | NR | 4 weeks | NR | NR | 8 weeks | Vivibot | Multimodal platform (via Facebook message) | Rule-based | Chatbot | Daily access | Text Emoji | Text  Emoji Image Video |
| Fulmer et al (2018) [27] | Cognitive restructuring Emotion identification  Journaling and relaxation strategies | 2 weeks  /4 weeks | NR | NR | None | Tess | Multimodal (via SMS or Slack or Facebook message) | AI-based  (ML, EA) | Chatbot | Daily access  /Biweekly access | Text | Text  Emoji |
| Fitzpatrick et al (2017) [45] | Challenging cognitive distortions Behavioral activation | 2 weeks | 20 | 90 seconds to 10 minutes | None | Woebot | Stand-alone desktop or mobile app | AI-based  (NLP) | Chatbot | Daily access | Text Emoji | Text  Emoji Video |
| Pinto et al (2016) [40] | Communication strategy | 12 weeks | 3 | 15 to 20 minutes | None | eSMART-MH | Stand-alone web/mobile system | Rule-based | Both | Once a month | Text | Text  Video |
| Burton et al (2016) [28] | Identifying and challenging negative cognitions Promoting positive reframing of thoughts  Behavioral activation exercises Reflect on common patterns of negative thinking  Encourages self-report of mood, sleep, and activities  Relaxation technique | 4 weeks | NR | NR | None | Help4Mood | Stand-alone computer system | AI-based  (EA） | Chatbot | Daily access | Text | Text  Image Voice |

ABBREVIATIONS: AI = artificial intelligence; SMS = Short Message Service; NR = not reported; ML = machine learning; NLP = natural language processing; DL = deep learning; EA = emotion algorithms.

**Table S6.** GRADE summary of findings.

| **No. of RCTs** | **No. of participants** | **Risk of bias** | **Inconsistency** | **Indirectness** | **Imprecision** | **Publication bias** | **Certainty** | **Importance** |
| --- | --- | --- | --- | --- | --- | --- | --- | --- |
| **Depressive symptoms (End of treatment)** | | | | | | | | |
| 27 | Chatbot group: 2147 Control group: 2100 | serious | serious | not serious | not serious | not serious | ⊕⊕○○ | CRITICAL |
| **Depressive symptoms (Follow up)** | | | | | | | | |
| 9 | Chatbot group: 615 Control group: 668 | serious | serious | not serious | not serious | not serious | ⊕⊕○○ | IMPORTANT |
| **Anxiety symptoms (End of treatment)** | | | | | | | | |
| 25 | Chatbot group: 2112 Control group: 2046 | serious | serious | not serious | not serious | not serious | ⊕⊕○○ | CRITICAL |
| **Anxiety symptoms (Follow up)** | | | | | | | | |
| 8 | Chatbot group: 566 Control group: 619 | serious | serious | not serious | serious | not serious | ⊕○○○ | IMPORTANT |

ABBREVIATIONS: RCT = randomized controlled trial
Note: Evidence from RCTs starts at high certainty. It was downgraded by one level (-1) for risk of bias due to the lack of participant and personnel blinding inherent to chatbot interventions. For inconsistency, evidence was downgraded by one level (-1) for moderate heterogeneity (*I*² > 50%). For imprecision, evidence was downgraded by one level (-1) if the 95% CI crossed the line of no clinical effect (zero).

**Table S7.** AIC/BIC-based multi-model inference of multivariable meta-regression for depressive and anxiety symptoms.

| **Model** | **Variable** | **Depressive symptoms** | | | | |  | **Anxiety symptoms** | | | | |
| --- | --- | --- | --- | --- | --- | --- | --- | --- | --- | --- | --- | --- |
|  |  | **R^2^ (%)** | **τ²** | ***I*^2^ (%)** | **AIC** | **BIC** |  | **R^2^ (%)** | **τ²** | ***I*^2^ (%)** | **AIC** | **BIC** |
| **Model 1: Sample/Clinical Characteristics** | Age Female proportion Participants | 4.26 | 0.10 | 74.36 | 34.50 | 40.79 |  | 39.52 | 0.02 | 39.57 | 9.23 | 14.91 |
| **Model 2: Technical Features** | Platform Chatbot type Intervention length | 12.97 | 0.09 | 71.63 | 34.87 | 42.42 |  | 57.11 | 0.01 | 32.57 | 13.31 | 20.12 |
| **Model 3: Study Design** | Control type  Country  ROB | 16.96 | 0.08 | 70.19 | 33.30 | 40.85 |  | 63.53 | 0.01 | 28.32 | 8.16 | 14.97 |

ABBREVIATIONS: AIC = Akaike Information Criterion; BIC = Bayesian Information Criterion; ROB = Risk of bias.

Notes: R^2^ represents the proportion of between-study variance explained by the model; τ² represents the estimated residual between-study variance; *I*^2^ represents the proportion of residual variance attributed to heterogeneity. Lower AIC and BIC values indicate a better balance between goodness-of-fit and model complexity.
