# Supplementary material for: Efficacy, User Engagement, and Acceptability of Cognitive Behavioral Therapy–Oriented Psychological Chatbots for Adults With Depressive and/or Anxiety Symptoms: Systematic Review and Meta-Analysis of Randomized Controlled Trials
Source: J Med Internet Res. 2026 May 8;28:e82677. doi: 10.2196/82677 (PMC13154727; doi:10.2196/82677)
Supplement: Multimedia Appendix 2 [file jmir-v28-e82677-s002.docx]

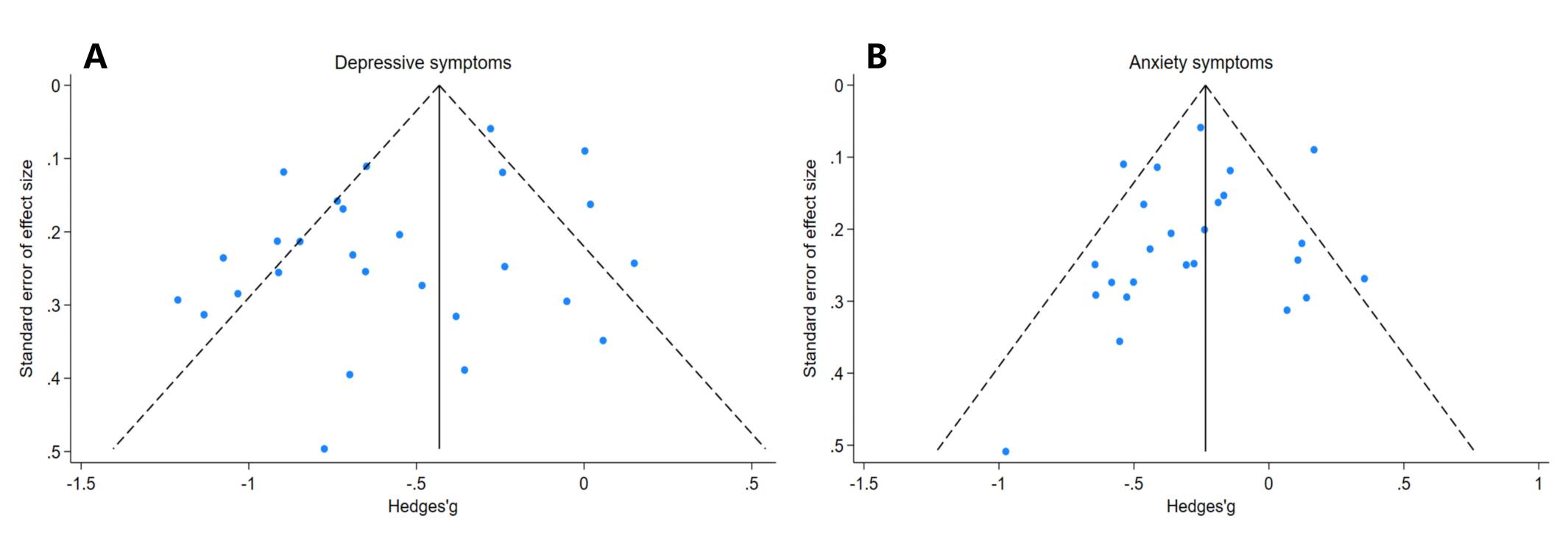


# **Figure S1. A funnel plot including studies of depressive (A) and anxiety symptoms (B).** Note: Bule dots represent individual studies, with the x - axis showing Hedges’ g (effect size) and the y - axis showing standard error (SE). The dashed lines indicate the expected distribution under no bias.


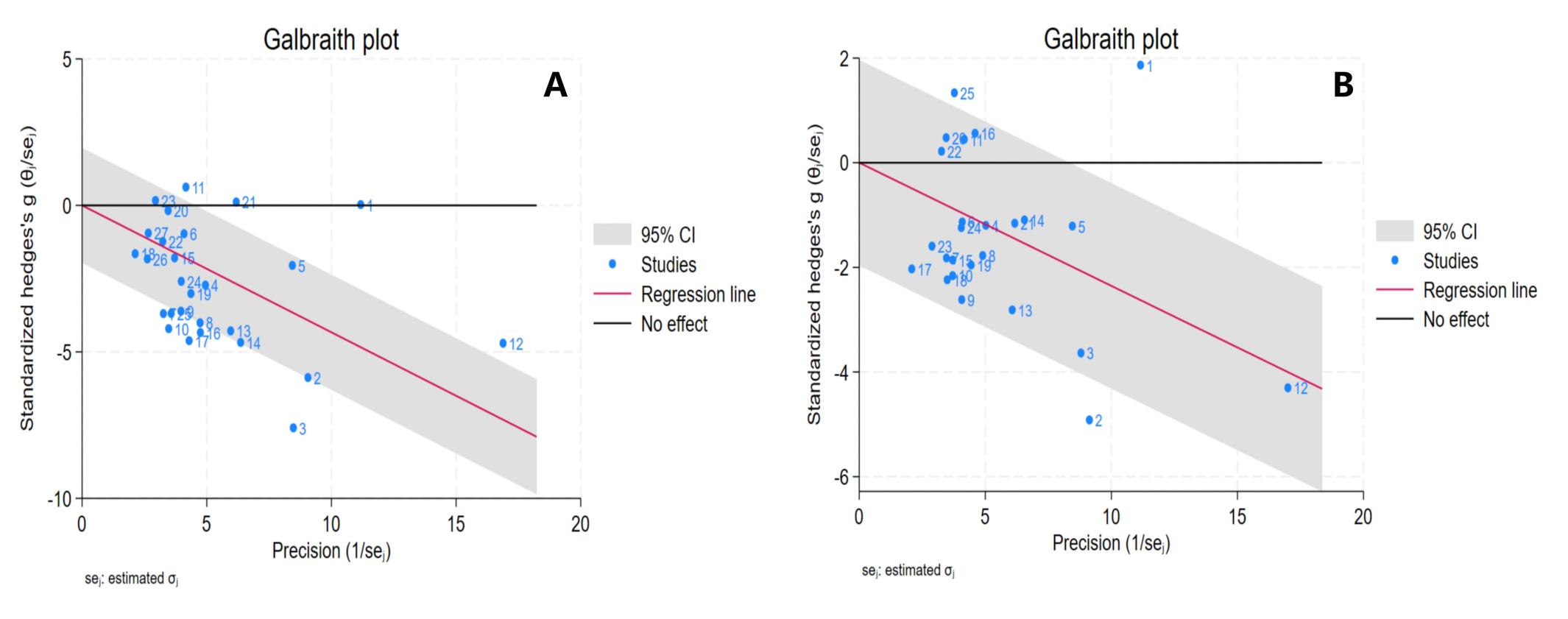


**Figure S2. A galbraith plot including studies of depressive (A) and anxiety symptoms (B).** Note: The x-axis shows the precision (inverse of the standard error, 1/SE), and the y-axis displays the standardized effect size (Hedges' g divided by its standard error,θ/SE). Each point represents an individual study. The solid line through the origin indicates the pooled effect size estimate from a fixed-effects model, with its slope corresponding to the overall effect. The dispersion of points around this line reflects the degree of between-study heterogeneity; points lying outside the expected confidence bands may indicate potential outliers or influential studies.


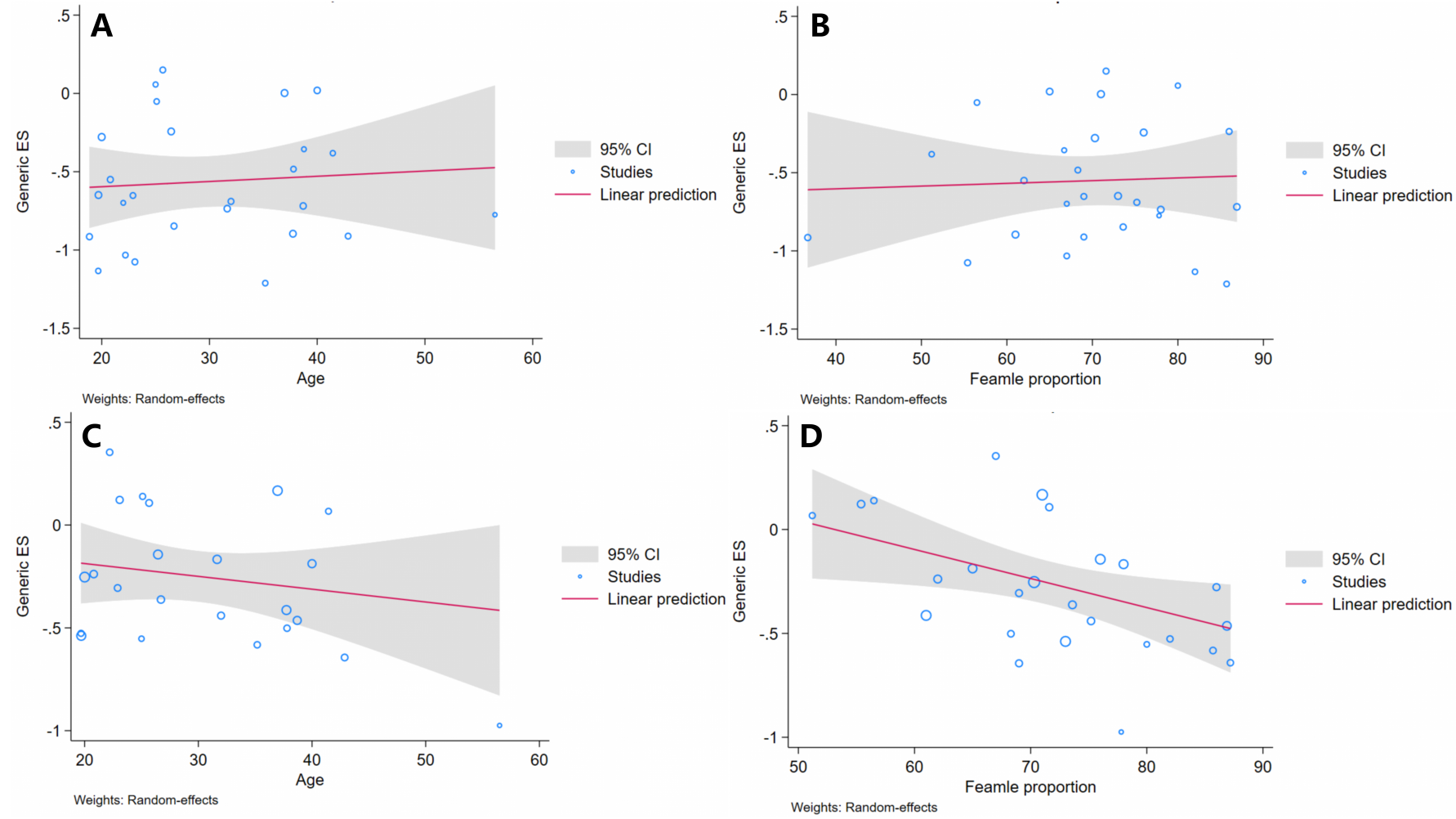


# **Figure S3. Meta-regression bubble plots of the effects of age and gender on depressive (A and B) and anxiety symptoms (C and D).** Note: The blue circles represent individual studies, circle size reflects study - specific weights.
